# Exploring the Relationship between Cognitive Performance, Performance-Based Functioning, and Everyday Functioning in People with Schizophrenia: a Systematic Literature Review

**DOI:** 10.1093/schbul/sbag121

**Published:** 2026-08-20

**Authors:** Philip D Harvey, Nina Schooler, Anthony O Ahmed

**Affiliations:** University of Miami Miller School of Medicine, Miami, FL 33136, United States; SUNY Downstate Health Sciences Center, Brooklyn, NY 11203, United States; Department of Psychiatry, Weill Cornell Medicine, New York, NY 10605, United States

**Keywords:** cognition, functional capacity, real-world outcomes, psychometric instruments

## Abstract

**Background and Hypothesis:**

Cognitive and functional impairments are core features of schizophrenia with considerable disease burden. However, the relationships between cognitive performance, functional capacity, and real-world functioning remain incompletely understood. This systematic review aimed to clarify the magnitude and nature of these interrelationships across primary research studies.

**Study Design:**

A systematic review (PROSPERO CRD42024532729) was conducted across MEDLINE, Embase, PsycINFO, and Cochrane Database. Eligible articles assessed cognitive performance, functional capacity, and real-world functioning in adults with schizophrenia using standard psychometric instruments, and reported quantitative relationships between ≥1 of these constructs. Due to substantial heterogeneity in study designs, outcome measures, and reported metrics, a narrative synthesis was performed.

**Study Results:**

Overall, 104 articles with unique datasets were included (88 observational, 16 interventional). The relationship between cognitive performance and functional capacity was the most studied (63/104 articles; 60.5%), with predominantly medium-to-large effect sizes. In contrast, associations between cognitive performance and real-world functioning were less frequently reported (43/104 articles, 41.3%) and largely small-to-medium, with only 2 large effect sizes identified. Associations between functional capacity and real-world functioning were variable (41/104 articles; 39.4%) and predominantly small-to-medium, with only 4 large effect sizes reported. Associations were less consistent when examined at the level of specific cognitive or functional domain.

**Conclusions:**

This review highlights the central role of cognition in shaping functional abilities, while reinforcing the complexity of translating these gains into meaningful improvements in everyday functioning. A multimodal, person-centered approach is needed to effectively translate cognitive improvements into meaningful real-world outcomes in people with schizophrenia.

## Introduction

Schizophrenia is associated with substantial morbidity and is the third largest contributor to the global burden of mental disorders and the 20th leading cause of overall years lived with disability worldwide.^1^ Cognitive impairment is a highly prevalent feature of schizophrenia, affecting more than 80% of individuals across the course of illness.^2–6^ Beyond an association with poor general and health-related quality of life, cognitive impairment is also an established risk factor for mortality from natural causes.^7–9^ Underpinning cognitive impairment in schizophrenia are deficits in specific neurocognitive domains, which include speed of processing, attention, working memory, verbal learning and memory, visual learning and memory, reasoning and problem solving; and social cognitive domains, such as emotional processing, social perception, attributional style, and emotional regulation.^10^^,^^11^

Functional disability is another core feature of schizophrenia, which includes impairments in independent, social, and role functioning.^12^^,^^13^ Assessment of functioning in schizophrenia typically distinguishes between two related constructs: functional capacity and real-world functioning.^14^ Functional capacity reflects an individual’s capacity to complete everyday activities and is assessed using standardized performance-based measures administered under controlled conditions. In contrast, real-world functioning reflects an individual’s performance of everyday activities in daily life.^14^

Although direct observation of real-world functioning can provide valuable insight into an individual’s actual performance, it is often limited by practical constraints. For example, the time and resources needed to fully capture functioning in real-world practice are not always feasible in routine research and clinical settings.^15^ As a result, proxy reports from caregivers with extensive contact are frequently used.^15^ However, these may not be feasible in all contexts, including those in assisted living facilities where proxy-patient contact is limited.^15^ In addition, real-world functioning is influenced by external factors, including the opportunity and personal motivation to perform activities. Therefore, performance-based measures of functional capacity are often preferred to gain insights into individual functioning and the impact of interventions as they provide a more controlled assessment of functional ability and are less impacted by these contextual influences.

Although cognitive performance and overall functioning in schizophrenia have been widely assessed using validated psychometric instruments,^16^^,^^17^ relatively few studies have examined the relationship between functional capacity and real-world functioning examined as distinct constructs.^18–24^ Of the systematic reviews and meta-analyses that have examined the relationship between cognitive performance and functioning in schizophrenia,^21^^,^^23–28^ few have explored the 3-domain relationship between cognitive performance, functional capacity, and real-world functioning with the exception of Kharawala et al.^22^

Given that real-world functioning is influenced by a broader range of factors than functional capacity, conflation of these constructs in research may distort estimates of association between cognitive performance and functioning in schizophrenia. Moreover, greater variability in function may be observed among individuals with comparable cognitive performance. A clear distinction between functional domains is therefore essential for a more nuanced understanding of these relationships, particularly in the context of cognition-targeted interventions.

A notable unmet need in schizophrenia is the absence of approved interventions targeting cognitive impairment, with no specific pharmacological treatments currently available. Furthermore, given the multifaceted nature of cognition as a primary endpoint in clinical trials for schizophrenia, the US Food and Drug Administration (FDA) mandates a co-primary endpoint of demonstrating meaningful functional improvement.^29^^,^^30^ In schizophrenia research, person-centered approaches such as those informed by the World Health Organization’s International Classification of Functioning (ICF) prioritize meaningful functioning by considering not only clinical symptoms but also daily activities and participation, available resources, and socioenvironmental factors.^5^ Therefore, comprehensively assessing the relationships between cognitive and functional domains could enhance understanding of the biopsychosocial model of schizophrenia and inform the development of integrated person-centered management strategies.

This systematic literature review addresses current gaps in previous findings on the relationship between cognitive performance, functional capacity, and real-world functioning by synthesizing evidence from original studies reporting quantitative relationships using validated psychometric instrument–defined scores. The aim of this systematic review was to characterize the magnitude of the relationships between (1) cognitive performance and functional capacity, (2) cognitive performance and real-world functioning, and (3) functional capacity and real-world functioning in individuals with schizophrenia across observational and interventional studies.

## Methodology

The systematic literature review was conducted in accordance with the Preferred Reporting Items for Systematic Reviews and Meta-Analysis (PRISMA) guidelines.^31^ The protocol was registered with PROSPERO on April 17, 2024 (CRD42024532729).^32^

### Search Strategy

The electronic databases MEDLINE, Embase, PsycINFO, and the Cochrane Database of Systematic Reviews were searched for relevant articles on April 17, 2024. The search strategy combined controlled vocabulary and keyword terms for the condition “schizophrenia spectrum disorder” and constructs of interest “cognitive performance”, “functional capacity”, and “real-world functioning”, including the psychometric instruments of interest listed in Table 1. No limits were placed on the year in which the article was published. No searches of grey literature sources were conducted. Conference abstracts and non-English language articles with English language abstracts were not included, considering that insufficient detail of analysis and results will be reported within an abstract for meaningful interpretation. Case reports and series were excluded. Reviews were excluded, but review articles relevant to the study questions were identified during screening and their reference lists were reviewed to identify further relevant articles. See Supplementary Tables 1–3 for the full search strategy, including the full search terms and number of hits.

**Table 1:** Psychometric Assessment Instruments Identified^*^ for each Construct

**Construct**	**Instrument**
**Cognitive performance**	MATRICS Consensus Cognitive Battery (MCCB); Brief Assessment of Cognition in Schizophrenia (BACS/BAC-App); Cambridge Neuropsychological Test Automated Battery (CANTAB); Mattis Dementia Rating Scale (DRS); Social Cognition Psychometric Evaluation (SCOPE) Social Cognition Tests; Cogstate Schizophrenia Battery (CSB); Repeatable Battery for the Assessment of Neuropsychological Status (RBANS)
**Functional capacity**	UCSD Performance-based Skills Assessment (UPSA); Schizophrenia Cognition Rating Scale (SCoRS); Virtual Reality Functional Capacity Assessment Tool (VRFCAT); Social Skills Performance Assessment (SSPA); Medication Management Ability Assessment (MMAA); Maryland Assessment of Social Competence (MASC); Cognitive Assessment Interview (CAI); Functional Skills Assessment and Training (FUNSAT)
**Real-world functioning**	Specific Levels of Functioning Scale (SLOF); Quality of Life Scale (QLS); Social Functioning Scale (SFS); Social Adjustment Scale (SAS); World Health Organization Disability Assessment Schedule (WHODAS) 2.0; Milestones: employment, vocational outcomes, stable relationships/marriage, independent living, college/adult education attendance

^*^Selection of instruments was informed by the expertise of the authors.

MATRICS, Measurement and Treatment Research to Improve Cognition in Schizophrenia (MATRICS) Consensus Cognitive Battery (MCCB); UCSD, University of California San Diego.

Following completion of the systematic literature review search, a limited number of additional references were identified for inclusion based on the expert knowledge of the authors. These publications were deemed highly relevant to the research objectives but were not captured by the pre-defined search strategy.

### Selection Process

The identified publications were screened at 2 levels based on the pre-defined eligibility criteria: (1) title and abstract screening and (2) full text screening. Before screening, all reviewers conducted a pilot filtering of 10% of articles, with any discrepancies discussed and resolved to ensure alignment and minimize heterogeneity when screening articles against the eligibility criteria. Screening of each abstract and full-text thereafter was conducted by two reviewers (blinded to each other’s decision) with reconciliation by discussion through a third reviewer. The software platform Nested Knowledge and dual reviewer screening were used to manage de-duplication of articles from multiple databases.

### Eligibility Criteria

The following inclusion criteria were developed according to the Participant, Intervention, Comparison, Outcomes, Study design framework.

#### Participants

Eligible studies included adults with a diagnosis of schizophrenia, schizophrenia spectrum disorders (SSDs), or first episode non-affective psychosis. In studies with populations comprising mixed diagnoses and age-groups, ≥75% of the population needed to be ≥18 years of age and have a diagnosis of schizophrenia or SSD, or report relevant data for a subgroup of adults with schizophrenia or SSD. Studies that included participants with a diagnosis of affective psychoses or substance-induced psychoses were excluded.

#### Intervention and Comparison

Both observational and interventional studies, with or without a comparator, were included. Studies were included regardless of intervention type, including pharmacological, cognitive training, or psychosocial interventions targeting cognitive performance or functioning.

#### Outcomes

The outcome constructs of interest were cognitive performance, functional capacity, and real-world functioning. The psychometric assessment instruments of interest for each outcome construct are presented in Table 1. Modified or brief versions of instruments, such as the University of California San Diego (UCSD) Performance-based Skills Assessment (UPSA)–Brief (UPSA-B), abbreviated Measurement and Treatment Research to Improve Cognition in Schizophrenia (MATRICS) Consensus Cognitive Battery (MCCB) neuropsychological battery and abbreviated Quality of Life Scale (QLS), were included. For inclusion, studies had to provide quantitative data on 1 or more of the following relationships, measured according to 1 or more of the instruments listed in Table 1:

- Cognitive performance and functional capacity
- Cognitive performance and real-world functioning
- Functional capacity and real-world functioning

Studies reporting directionality or statistical significance without explicit quantitative estimates were also considered for inclusion when such data contributed meaningfully to the synthesis.

#### Study Design

Any type of observational or interventional study reporting the outcomes of interest was included. A minimum sample size of at least 30 individuals in the relevant analyses was required for inclusion to allow for meaningful interpretation of the resulting correlational findings.

#### Overlapping Datasets

Overlapping datasets and aggregated articles were identified during data extraction, where possible. Articles with subset analyses (re-analyses or preliminary publications) and no unique analyses of datasets from the original or final publication were excluded. A list of overlapping publications and the rationale for inclusion of a single representative publication per dataset is provided in Supplementary Table 4. A total of 13 common study cohorts were identified across 36 publications. Of the 36 publications, 21 had interim data analyses or no unique analyses of common datasets and were thus excluded prior to data synthesis. For Miller et al.,^33^ a unique analysis of unpublished data was obtained directly from the study authors (see **Supplementary materials** for details of the analysis).

### Data Synthesis

The following details were extracted for all studies in Excel: year of publication, publication type, study objective(s), sample size, population, outcome constructs of interest examined, psychometric instruments used to measure each construct of interest, and quantitative data on the relationship between (1) cognitive performance and functional capacity, (2) cognitive performance and real-world functioning, and (3) functional capacity and real-world functioning. Data were extracted by a single reviewer using a standardized extraction table and independently checked by a second reviewer. A pilot extraction of the first 3 articles was performed to ensure accuracy before proceeding with the full review. All extracted fields were cross-checked by the second reviewer and any discrepancies were resolved through discussion and, where required, consultation with a third reviewer.

When studies reported quantitative relationship data derived from global, total, or interviewer scores on psychometric instruments, only these data were extracted. When no overall score data were available, relationship data derived from subscale or domain-specific scores were extracted. Psychometric instruments assessing cognitive performance were considered measures of “global cognition” unless described otherwise. For example, a modified MCCB that excluded social cognition was considered a measure of neurocognition. For interventional studies, additional details were extracted, including the name, description, and duration of the intervention and comparator, if applicable. Where sufficient data were reported, relationship effect sizes were interpreted in accordance with Cohen’s guidelines: large (r ≥ 0.5; d ≥ 0.8), medium (0.3 ≤ r < 0.5; 0.5 ≤ d < 0.8), small (0.1 ≤ r < 0.3; 0.20 ≤ d < 0.5), or minimal (r < 0.10; d < 0.2).^34^ Individual studies could contribute to more than 1 category if they assessed multiple psychometric instrument pairs or outcomes. No analysis by subgroup or subset was conducted.

Due to substantial heterogeneity in the outcome measures, psychometric instruments, and reported metrics of association, studies were not directly comparable. In addition, variation in study design and non-independence of datasets precluded valid pooling. Therefore, a narrative synthesis was deemed the most appropriate methodological approach and was conducted to include descriptions of the unique studies identified, and the findings with respect to the quantitative relationships between constructs.

### Quality Assessment

Due to the diversity of study designs, outcome measures and reporting methods expected among included studies, it was determined that employing a single standardized risk-of-bias assessment tool across all included studies would not be appropriate. Similarly, utilizing multiple tools across all included studies may have compromised consistency and comparability. Instead, several stringent inclusion criteria were applied to ensure methodological quality (sample size: ≥30 participants; age: ≥18 years; diagnosis: schizophrenia, SSD, or first episode non-affective psychosis; publication type: exclusion of case reports, conference abstracts, and articles lacking sufficient methodological detail) by identifying studies with data for meaningful interpretation. Most of the studies included were cross-sectional, with fewer longitudinal or interventional studies, and there was variability in adjustment for potential confounders. These factors should be considered in the assessment of the strength and generalizability of the reported associations.

## Results

### Literature Search Results

**Figure 1:**
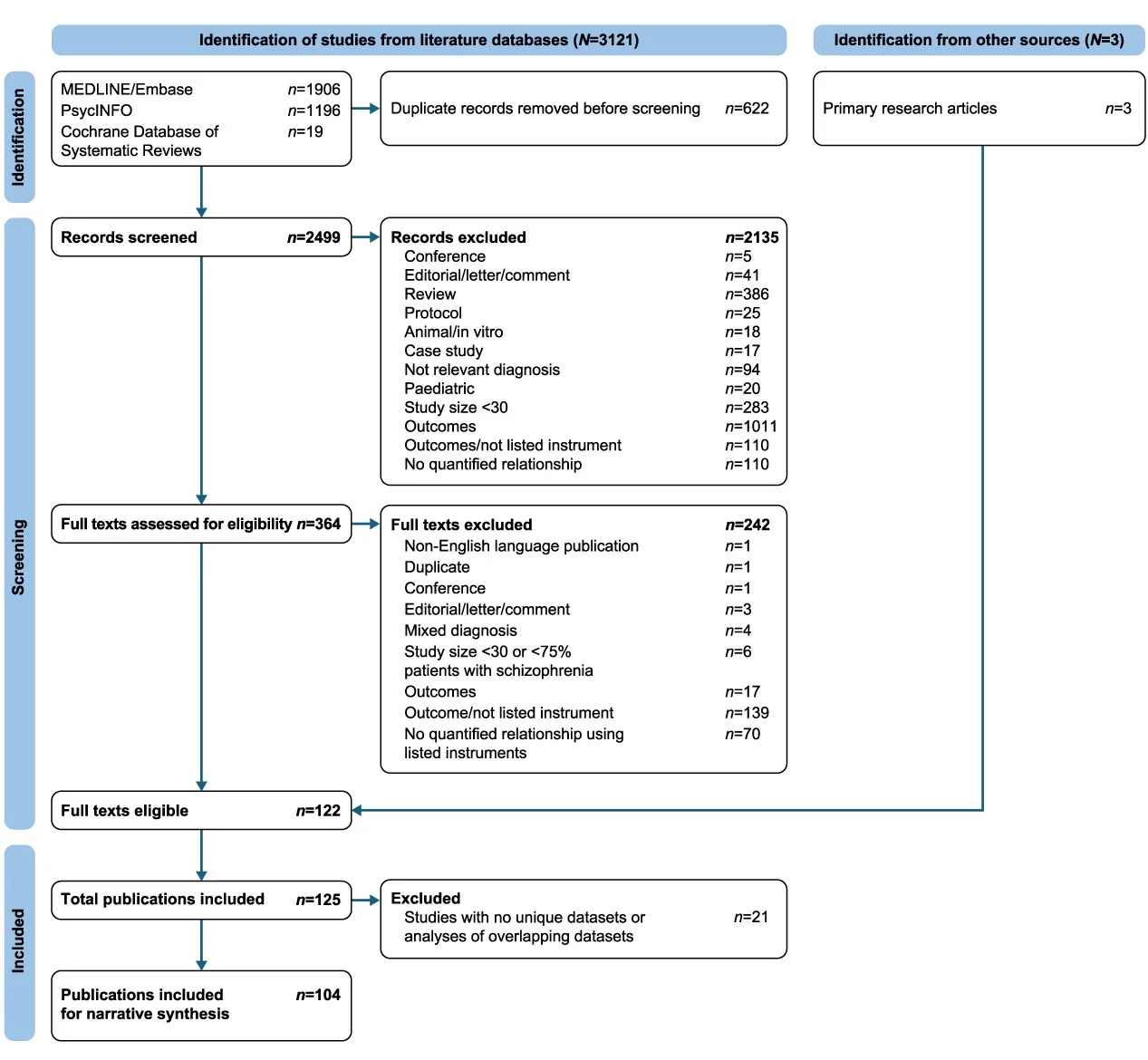
PRISMA Flow Diagram for Search Results

A total of 2499 citations were identified from literature databases (MEDLINE, Embase, PsycINFO, Cochrane Database of Systematic Reviews) after de-duplication of common entries across databases (Figure 1). A further 3 articles were identified and approved for inclusion by all authors.^33^^,^^35^^,^^36^ After abstract screening, full texts were reviewed, and 125 primary publications providing quantitative data on the relationship between at least 2 outcome constructs measured by the instruments listed in Table 1, were included. Following the exclusion of publications with overlapping study cohorts and no unique analyses, a final 104 articles covering 104 unique studies were included in the narrative synthesis.

### Overview of Included Studies

Supplementary Tables 5–10 provide a summary of the key characteristics of the 104 articles included in the narrative synthesis for the relationship between cognitive performance and functional capacity (Supplementary Tables 5 and 6), the relationship between cognitive performance and real-world functioning (Supplementary Tables 7 and 8), and the relationship between functional capacity and real-world functioning (Supplementary Tables 9 and 10). Articles were published between 1999 and 2024. The largest proportion of studies included individuals from the United States (*n* = 53, 51.0%), Japan (*n* = 12, 11.5 %), and Italy (*n* = 7, 6.7%). A greater number of observational studies (*n* = 88, 84.6%) were included than interventional studies (*n* = 16, 15.4%). Of the observational studies, most had a cross-sectional design (*n* = 77, 87.5%) and 10 (11.4%) were longitudinal. The 16 interventional studies examined pharmacological, cognitive training, or psychosocial interventions and were predominantly randomized controlled trials (*n* = 12, 75.0%).

Of the 104 publications, over half reported quantitative data on the relationship between cognitive performance and functional capacity (*n* = 63, 60.5%) (Supplementary Table 5),^30^^,^^33^^,^^35–95^ and over one-third reported quantitative data on the relationship between cognitive performance and real-world functioning (*n* = 43, 41.3%) (Supplementary Table 7)^33^^,^^36^^,^^37^^,^^39–42^^,^^44^^,^^46^^,^^56–58^^,^^61^^,^^63^^,^^67^^,^^69^^,^^70^^,^^73^^,^^75^^,^^76^^,^^81^^,^^96–117^ and between functional capacity and real-world functioning (*n* = 41, 39.4%) (Supplementary Table 9).^30^^,^^33^^,^^37^^,^^40–44^^,^^46^^,^^52^^,^^58^^,^^61^^,^^63^^,^^65^^,^^73^^,^^75–77^^,^^81^^,^^99^^,^^100^^,^^108^^,^^118–136^ Quantitative data on all 3 relationships were reported by 14 (13.5%) studies^33^^,^^37^^,^^40–42^^,^^44^^,^^46^^,^^58^^,^^61^^,^^63^^,^^73^^,^^75^^,^^76^^,^^81^; all were observational. A total of 65 (62.5%) studies reported relevant effect size data for interpretation of the magnitude of at least one relationship (Supplementary Tables 6,8, and 10).^30^^,^^33^^,^^36–40^^,^^42^^,^^43^^,^^45^^,^^47–49^^,^^52–57^^,^^59^^,^^62–67^^,^^69–73^^,^^75–80^^,^^82–85^^,^^88–91^^,^^97^^,^^99–101^^,^^103^^,^^108–111^^,^^119^^,^^122^^,^^124–128^^,^^134–137^ Correlation analyses were the most frequently reported analysis type used to explore relationships between variables, with or without subsequent regression analyses.

### Characteristics of Study Populations

Across included studies, the sample size of individuals with schizophrenia ranged from 35 to 4310 (median [interquartile range, IQR]: 114 [61.8–187.3]) for observational studies and from 30 to 488 (median [IQR]: 107 [80.3–133.0]) for interventional studies. Sample size ranges were 35–4310 (observational) and 69–488 (interventional) for studies examining the relationship between cognitive performance and functional capacity, 36–921 (observational) and 32–136 (interventional) for studies examining the relationship between cognitive performance and real-world functioning, and 45–921 (observational) and 30–54 (interventional) for studies examining the relationship between functional capacity and real-world functioning. Most studies included individuals with a diagnosis of schizophrenia or schizoaffective disorder only (*n* = 85, 81.7%: observational, *n* = 72; interventional, *n* = 13) and from a community/outpatient/day hospitalization clinical setting (*n* = 73, 70.2%: observational, *n* = 61; interventional, *n* = 12).

### Narrative Synthesis

#### Relationship between Cognitive Performance and Functional Capacity

Sixty-three studies (observational and interventional) provided quantitative data on the association between scores for cognitive performance and functional capacity as measured by at least 1 set of paired psychometric instruments (Supplementary Table 5). Of these, 46 studies provided sufficient data for effect size interpretation. Effect sizes for the relationship between overall instrument scores were reported across 62 pairings (Table 2); the most frequent pairing was MCCB and UPSA/UPSA-B. A large effect size was found across 38 pairings, a medium effect size across 17 pairings, and a small-minimal effect size across 9 pairings.

**Table 2:** Strength of the Relationship between Cognitive Performance and Functional Capacity Overall Instrument Scores by Instrument Pairs (*n* = 63)

**Instrument pair**			**Relationship between overall instrument scores**				
				**Strength**			
**Cognitive performance**	**Functional capacity**	**No. studies**	**No. studies with relevant effect size data**	**Minimal** **r < 0.10** **d < 0.2**	**Small** **0.1 ≤ r < 0.3 0.20 ≤ d < 0.5**	**Medium** **0.3 ≤ r < 0.5 0.5 ≤ d < 0.8**	**Large** **r ≥ 0.5** **d ≥ 0.8**
MCCB	UPSA/UPSA-B	40	27	0	0	6	22
MCCB	CAI	8	7	0	2	1	4
MCCB	SSPA	8	5	0	2	3	0
BACS	UPSA/UPSA-B	6	5	0	0	2	3
BACS	SCoRS	6	5	0	1	1	2
MCCB	SCoRS	4	4	0	1	1	2
MCCB	VRFCAT	2	2	0	0	0	2
MCCB	MASC	2	2	0	1	1	0
DRS	SSPA	2	2	0	0	1	1
CogState	UPSA/UPSA-B	2	1	0	0	0	1
DRS	UPSA	2	1	0	0	0	1
SCOPE	SSPA	1	1	1	1	1	0
		Total across pairings^*^	62	1	8	17	38

^*^Studies may have evaluated more than one instrument pairing.

BACS, Brief Assessment of Cognition in Schizophrenia; CAI, Cognitive Assessment Interview; CogState, CogState Schizophrenia Battery; DRS, Dementia Rating Scale; MASC, Maryland Assessment of Social Competence; MCCB, MATRICS Consensus Cognitive Battery; MMAA, Multimodal Attention Assessment Battery; SCOPE, Social Cognition Psychometric Evaluation; SCoRS, Schizophrenia Cognition Rating Scale; SSPA, Social Skills Performance Assessment; UPSA, UCSD Performance-Based Skills Assessment; UPSA-B, UCSD Performance-Based Skills Assessment—Brief version; VRFCAT, Virtual Reality Functional Capacity Assessment Tool.

#### Observational Studies

Of the 88 observational studies, 54 (61.4%) provided quantitative data on the relationship between cognitive performance and functional capacity (Supplementary Table 5).^30^^,^^33^^,^^35–85^^,^^95^ The MCCB and UPSA were the most frequently used psychometric instruments in observational studies to assess cognitive performance and functional capacity, respectively, either alone or in combination. Other common psychometric instrument pairings included the MCCB with the Cognitive Assessment Interview (CAI), and the Brief Assessment of Cognition in Schizophrenia (BACS) with the UPSA. The majority of observational studies provided quantitative data on the association between scores for cognitive performance and functional capacity as measured by 1 set of paired psychometric instruments^35^^,^^37–41^^,^^44–51^^,^^55^^,^^58–63^^,^^65^^,^^68–76^^,^^81–83^^,^^85^^,^^95^; 20 studies measured the relationship with at least 2 instrument sets.^30^^,^^33^^,^^36^^,^^42–44^^,^^52–54^^,^^56^^,^^57^^,^^63^^,^^64^^,^^66^^,^^67^^,^^77–80^^,^^84^ A total of 42 studies provided sufficient data for effect size interpretation.^30^^,^^33^^,^^36–40^^,^^42^^,^^43^^,^^45^^,^^47–49^^,^^52–57^^,^^59^^,^^62–67^^,^^69–73^^,^^75–80^^,^^82–85^^,^^137^

**Associations by overall instrument scores.** Forty-eight observational studies provided quantitative data on the relationship between overall instrument scores for cognitive performance and functional capacity (Supplementary Table 5).^30^^,^^33^^,^^35^^,^^37–46^^,^^48–50^^,^^52–55^^,^^57–66^^,^^70–85^^,^^137^^,^^138^ A total of 43 studies found a significant positive association between better cognitive performance and greater functional capacity based on at least 1 set of instrument scores.^33^^,^^37–42^^,^^45^^,^^46^^,^^48–50^^,^^52–55^^,^^57–59^^,^^61–66^^,^^70–85^^,^^137^^,^^138^ One study^60^ found a non-significant relationship and the remaining 4 studies lacked sufficient data to determine the statistical significance of the association.^30^^,^^35^^,^^43^^,^^44^ A total of 38 studies provided effect size estimates for at least 1 set of instruments assessing cognitive performance and functional capacity (Table 2).^30^^,^^33^^,^^37–40^^,^^42^^,^^43^^,^^45^^,^^48^^,^^49^^,^^52–54^^,^^57^^,^^59^^,^^62^^,^^64–66^^,^^70–73^^,^^75–80^^,^^82–85^^,^^95^ Of these, 26 found a large association,^30^^,^^39^^,^^40^^,^^42^^,^^43^^,^^48^^,^^49^^,^^52–55^^,^^57^^,^^59^^,^^62–66^^,^^73^^,^^75^^,^^76^^,^^78–80^^,^^82^^,^^85^ 19 a medium association,^33^^,^^37^^,^^43^^,^^45^^,^^52^^,^^54^^,^^57^^,^^63^^,^^64^^,^^70–72^^,^^77^^,^^78^^,^^83–85^^,^^95^ 7 a small association,^30^^,^^38^^,^^43^^,^^53^^,^^77^^,^^84^^,^^85^ and 1 a minimal association between at least 1 set of instruments for cognitive performance and functional capacity.^54^ Associations were generally consistent between the different instrument sets used within each study reporting relationship data for at least 2 instrument sets,^30^^,^^33^^,^^36^^,^^42–44^^,^^52–54^^,^^56^^,^^57^^,^^63^^,^^64^^,^^66^^,^^67^^,^^78–80^^,^^84^ with the exception of 1 study.^77^ For Ventura et al.,^77^ a small association was observed between the MCCB and functional capacity, which was significant when functional capacity was assessed using the CAI, but not the Schizophrenia Cognition Rating Scale (SCoRS). Of the instrument sets assessed, the MCCB and UPSA was the most common pairing and provided larger associations than other instrument pairs assessed within each study.^30^^,^^33^^,^^42^^,^^43^^,^^53^^,^^54^^,^^57^^,^^64^^,^^66^^,^^78–80^^,^^84^

**Associations by subtest or domain scores.** Six observational studies provided quantitative data on the relationship between cognitive performance and functional capacity exclusively in terms of subtest or domain-specific scores (Supplementary Table 5).^36^^,^^47^^,^^51^^,^^67–69^ Subtest scores were reported for the MCCB (*n* = 3), Social Cognition Psychometric Evaluation (SCOPE) Social Cognition Tests (*n* = 2), UPSA, (*n* = 2), and BACS (*n* = 1). Four studies reported relevant effect size data on the association between at least 1 set of instruments for cognitive performance and functional capacity (Supplementary Tables 5 and 6).^36^^,^^47^^,^^67^^,^^69^ Three studies reported an association between cognitive subtest scores and an overall score for functional capacity,^36^^,^^67^^,^^69^ while 1 study reported on the association between BACS total score and UPSA-B domain scores.^47^ In the few studies that reported effect size data for cognitive domains, the domains of speed of processing, working memory, and verbal learning showed the most consistent associations with functional capacity, whereas other domains demonstrated more limited and heterogeneous findings.^36^^,^^67^^,^^69^

#### Interventional Studies

Of the 16 interventional studies, 9 (56.3%) provided quantitative data on the relationship between cognitive performance and functional capacity (Supplementary Table 5).^86–94^ The MCCB and UPSA were the most frequently used psychometric instruments in interventional studies to assess cognitive performance and functional capacity, respectively, either alone or in combination. A total of 4 studies provided sufficient data for effect size interpretation.^88–91^

All 9 studies provided quantitative data on the relationship between overall instrument scores for cognitive performance and functional capacity (Supplementary Table 5).^86–94^ Eight of the 9 studies reported a statistically significant relationship based on at least 1 set of instrument scores, indicating that better cognitive performance was positively associated with greater functional capacity in individuals with schizophrenia.^86–92^^,^^94^ Of the 4 studies reporting relevant effect size data (Table 2), all assessed the association between the MCCB and UPSA/UPSA-B.^88–91^ Two studies found a large association between the MCCB and UPSA/UPSA-B^88^^,^^90^ and 1 found a medium association,^91^ while Marx et al.^89^ found a medium-to-large association for the 6-domain MCCB and a large association for the 7-domain MCCB. Seven studies assessed longitudinal associations.^86^^,^^87^^,^^89–91^^,^^93^^,^^94^ All except Tan et al.^93^ demonstrated a significant positive association between changes in cognitive performance and changes in functional capacity, with Tan et al.^93^ finding a non-significant association. The studies reporting sufficient effect size data found medium-to-large positive associations.^88–91^ No study reported a significant difference in the associations between treatment groups and findings were mixed regarding whether the strength of the relationship changed over time.^87^^,^^89^ No study reported associations by subtest or domain scores (Supplementary Table 5).

### Relationship between Cognitive Performance and Real-World Functioning

Forty-three studies (observational and interventional) provided quantitative data on the association between scores for cognitive performance and real-world functioning as measured by at least 1 set of paired psychometric instruments (Supplementary Table 7). Of these, 23 studies provided sufficient data for effect size interpretation. Effect sizes for the relationship between overall instrument scores were reported across 15 pairings (Table 3). A large effect size was found in 2 pairings, a medium effect size across 3 pairings, and a small-minimal effect in 10 pairings.

**Table 3:** Strength of the Relationship between Cognitive Performance and Real-World Functioning Overall Instrument Scores across Instrument Pairs (*n* = 43)

**Instrument pair**			**Relationship between overall instrument scores**				
				**Strength**			
**Cognitive performance**	**Real-world functioning**	**No. studies**	**No. studies with relevant effect size data**	**Minimal** **r < 0.10 d < 0.2**	**Small** **0.1 ≤ r < 0.3 0.20 ≤ d < 0.5**	**Medium** **0.3 ≤ r < 0.5 0.5 ≤ d < 0.8**	**Large** **r ≥ 0.5 d ≥ 0.8**
MCCB	SLOF	13	3	0	2	0	1
BACS	QLS	5	4	1	1	2	0
MCCB	QLS	4	1	0	1	0	0
MCCB	SFS	3	3	1	1	1	0
SCOPE	SLOF	3	0	0	0	0	0
MCCB	WHODAS	2	2	0	2	0	0
BACS	SFS	2	1	0	1	0	0
BACS	SLOF	1	1	0	0	0	1
		Total across pairings^*^	15	2	8	3	2

^*^Studies may have evaluated more than one instrument pairing.

BACS, Brief Assessment of Cognition in Schizophrenia; MCCB, MATRICS Consensus Cognitive Battery; QLS, Quality of Life Scale; SCOPE, Social Cognition Psychometric Evaluation; SFS, Social Functioning Scale; SLOF, Specific Levels of Functioning Scale; WHODAS, World Health Organization Disability Assessment Schedule.

#### Observational Studies

Of the 88 observational studies, 38 (43.2%) provided quantitative data on the relationship between cognitive performance and real-world functioning (Supplementary Table 7).^33^^,^^36^^,^^37^^,^^39–42^^,^^44^^,^^46^^,^^56–58^^,^^61^^,^^63^^,^^67^^,^^69^^,^^70^^,^^73^^,^^75^^,^^76^^,^^81^^,^^96–111^^,^^117^ The MCCB and Specific Levels of Functioning Scale (SLOF) were the most frequently used psychometric tools to assess cognitive performance and real-world functioning, respectively, either alone or in combination. Most studies provided quantitative data on the association between scores for cognitive performance and real-world functioning as measured by 1 set of paired psychometric instruments^33^^,^^37^^,^^39–42^^,^^46^^,^^57^^,^^58^^,^^61^^,^^63^^,^^69^^,^^70^^,^^75^^,^^76^^,^^81^^,^^96–98^^,^^100–111^^,^^117^; 4 studies measured the relationship with at least 2 instrument sets.^36^^,^^56^^,^^67^^,^^73^ A total of 23 studies provided sufficient data for effect size interpretation.^33^^,^^36^^,^^37^^,^^39^^,^^40^^,^^42^^,^^56^^,^^57^^,^^63^^,^^67^^,^^69^^,^^70^^,^^73^^,^^75^^,^^76^^,^^97^^,^^100^^,^^101^^,^^103^^,^^108–111^

**Associations by overall instrument scores.** Seventeen studies provided quantitative data on the association between overall instrument scores for cognitive performance and real-world functioning (Supplementary Table 7).^37^^,^^40^^,^^44^^,^^57^^,^^63^^,^^70^^,^^73^^,^^76^^,^^97^^,^^100^^,^^101^^,^^104^^,^^105^^,^^108–111^ Eight of the 17 studies showed a significant positive association between better cognitive performance and greater real-world functioning based on at least 1 set of instrument scores.^37^^,^^40^^,^^63^^,^^73^^,^^76^^,^^104^^,^^109^^,^^111^ The 9 remaining studies reported either no, or a numerical albeit non-significant relationship.^44^^,^^57^^,^^70^^,^^97^^,^^100^^,^^101^^,^^105^^,^^108^^,^^110^ Fourteen studies provided relevant effect size data on the association between at least 1 set of instruments for cognitive performance and real-world functioning (Table 3).^37^^,^^40^^,^^57^^,^^63^^,^^70^^,^^73^^,^^76^^,^^97^^,^^100^^,^^101^^,^^108–111^ Of these, 2 found a large association,^73^^,^^76^ 3 a medium association,^40^^,^^108^^,^^109^ 7 a small association,^37^^,^^57^^,^^63^^,^^73^^,^^100^^,^^101^^,^^110^ 2 a minimal association,^70^^,^^108^ and 1 with no association^97^ between at least 1 set of instruments for cognitive performance and real-world functioning. For the study that assessed the relationship by overall scores for at least 2 instrument sets,^73^ findings were mixed. Sumiyoshi et al.^73^ found a large significant association with the BACS when real-world functioning was assessed using the SLOF, but a small non-significant association when real-world functioning was assessed using the Social Functioning Scale (SFS).

**Associations by subtest or domain scores.** Twenty-one studies provided quantitative data on the relationship between cognitive performance and real-world functioning only in terms of subtest or domain-specific scores (Supplementary Table 7).^33^^,^^36^^,^^39^^,^^41^^,^^42^^,^^46^^,^^56^^,^^58^^,^^61^^,^^67^^,^^69^^,^^75^^,^^81^^,^^96^^,^^98^^,^^99^^,^^102^^,^^103^^,^^106^^,^^107^^,^^117^ Twelve studies reported subtest or subscale scores for the MCCB (*n* = 5), SCOPE (*n* = 2), SLOF (*n* = 7), and Social Adjustment Scale (*n* = 1).^36^^,^^41^^,^^42^^,^^46^^,^^56^^,^^61^^,^^67^^,^^69^^,^^99^^,^^102^^,^^106^^,^^107^ Three studies found a significant positive association between cognitive performance and real-world functioning for all examined subtests.^42^^,^^102^^,^^106^ Two studies found no significant association between cognitive performance and real-world everyday life skills, interpersonal relationships, or work skills.^41^^,^^46^ The remaining 7 studies showed mixed findings, where some subtests showed a significant association with cognitive performance or real-world functioning, but others did not.^36^^,^^56^^,^^61^^,^^67^^,^^69^^,^^99^^,^^107^ Relevant effect size data on the association between at least 1 set of instrument tests/subtests for cognitive performance and real-world functioning were reported by 5 studies (Supplementary Table 7; Supplementary Table 8).^36^^,^^42^^,^^56^^,^^67^^,^^69^ Of these, 3 studies found a medium association,^36^^,^^42^^,^^67^ all 5 found a small association,^36^^,^^42^^,^^56^^,^^67^^,^^69^ and 3 found a minimal association^36^^,^^67^^,^^69^; no studies found a large association. Across studies, no single cognitive domain showed a consistently strong association with real-world functioning at the subtest level, with most associations ranging from minimal to medium, reflecting greater variability in this relationship compared with that observed for functional capacity.

Nine of the 21 studies reported quantitative relationship data based on real-world milestone scores for residential status (*n* = 6),^33^^,^^58^^,^^75^^,^^81^^,^^103^^,^^117^ employment (*n* = 4),^39^^,^^96^^,^^98^^,^^117^ relationship status (*n* = 1),^117^ and financial independence (*n* = 2) (Supplementary Table 7).^33^^,^^81^ Seven studies found a significant association between cognitive performance and a real-world milestone, whereby poorer cognitive performance was associated with significantly worse residential status (*n* = 3),^33^^,^^75^^,^^103^ employment (*n* = 4),^39^^,^^96^^,^^98^^,^^117^ relationship status (*n* = 1),^117^ and financial independence (*n* = 1).^33^ Four studies reported relevant effect size data,^33^^,^^39^^,^^75^^,^^103^ with 2 finding a medium significant association between poorer cognitive performance and worse residential status,^75^^,^^103^ 1 a small significant association between poorer cognitive performance and unemployment,^39^ and another a small significant difference in cognitive performance by both residential and financial independence (Supplementary Table 7).^33^

#### Interventional Studies

Of the 16 interventional studies, 5 (31.3%) provided quantitative data on the relationship between cognitive performance and real-world functioning (Supplementary Table 7).^112–116^ The BACS and QLS were the most frequently used psychometric tools to assess cognitive performance and real-world functioning, respectively.

Two studies provided quantitative data on the association between overall instrument scores for cognitive performance and real-world functioning but did not show a significant association between these constructs (Supplementary Table 7).^112^^,^^116^ One study reported quantitative data on the relationship between the QLS measure of real-world functioning and MCCB subtests for speed of processing and cognitive control, revealing significant positive longitudinal associations.^113^ Two studies reported quantitative data on the relationship between cognitive performance and real-world milestones for employment^114^^,^^115^ but only John et al.^114^ found a significant association. No interventional study provided sufficient data for effect size assessment.

### Relationship between Functional Capacity and Real-World Functioning

Forty-one studies (observational and interventional) provided quantitative data on the association between scores for functional capacity and real-world functioning as measured by at least 1 set of paired psychometric instruments (Supplementary Table 9). Of these, 26 studies provided sufficient data for effect size interpretation. Effect sizes for the relationship between overall instrument scores were reported across 27 pairings (Table 4). A large effect size was found in 4 pairings, a medium effect size across 6 pairings, and a small effect in 19 pairings.

**Table 4:** Strength of the Relationship between Functional Capacity and Real-World Functioning Overall Instrument Scores across Instrument Pairs (*n* = 41)

**Instrument pair**				**Relationship between overall instrument scores**			
				**Strength**			
**Functional capacity**	**Real-world functioning**	**No. studies**	**No. studies with relevant effect size data**	**Minimal r < 0.10 d < 0.2**	**Small 0.1 ≤ r < 0.3 0.20 ≤ d < 0.5**	**Medium 0.3 ≤ r < 0.5 0.5 ≤ d < 0.8**	**Large r ≥ 0.5 d ≥ 0.8**
UPSA/UPSA-B	SLOF	16	5	0	4	1	1
SSPA	SLOF	7	2	0	2	0	0
SCoRS	SFS	4	3	0	2	0	1
UPSA/UPSA-B	QLS	3	2	0	2	0	0
CAI	SFS	2	2	0	1	1	1
UPSA/UPSA-B	SFS	2	2	0	0	2	0
VRFCAT	SLOF	2	2	0	2	0	0
SCoRS	QLS	2	2	0	0	2	0
UPSA	WHODAS	1	1	0	1	0	0
MASC	Global functional outcome	1	1	0	1	0	0
CAI	QLS	1	1	0	1	0	0
SCoRS	SLOF	1	1	0	0	0	1
SSPA	SAS	1	1	0	1	0	0
UPSA	Global functional outcome	1	1	0	1	0	0
MMAA	SLOF	1	1	0	1	0	0
		Total across pairings^*^	27	0	19	6	4

^*^Studies may have evaluated more than one instrument pairing.

CAI, Cognitive Assessment Interview; MASC, Maryland Assessment of Social Competence; MMAA, Multimodal Attention Assessment Battery; QLS, Quality of Life Scale; SAS, Simpson-Angus Scale; SCoRS, Schizophrenia Cognition Rating Scale; SFS, Social Functioning Scale; SLOF, Specific Level of Functioning scale; SSPA, Social Skills Performance Assessment; UPSA, UCSD Performance-Based Skills Assessment; UPSA-B, UCSD Performance-Based Skills Assessment—Brief version; VRFCAT, Virtual Reality Functional Capacity Assessment Tool; WHODAS, World Health Organization Disability Assessment Schedule.

#### Observational Studies

Of the 88 observational studies, 39 (44.4%) provided quantitative data on the relationship between functional capacity and real-world functioning (Supplementary Table 9).^30^^,^^33^^,^^37^^,^^40–44^^,^^46^^,^^52^^,^^58^^,^^61^^,^^63^^,^^65^^,^^73^^,^^75–77^^,^^81^^,^^99^^,^^100^^,^^108^^,^^118–134^ The UPSA and SLOF were the most frequently used psychometric tools to assess functional capacity and real-world functioning, respectively, either alone or in combination. Most studies provided quantitative data on the association between scores for functional capacity and real-world functioning as measured by 1 set of paired psychometric instruments^37^^,^^40–42^^,^^46^^,^^58^^,^^61^^,^^63^^,^^65^^,^^75^^,^^76^^,^^81^^,^^100^^,^^108^^,^^119^^,^^123–134^; 13 studies measured the relationship with at least 2 instrument sets.^30^^,^^33^^,^^43^^,^^44^^,^^52^^,^^63^^,^^73^^,^^77^^,^^99^^,^^118^^,^^120–122^ A total of 24 studies provided sufficient data for effect size interpretation.^30^^,^^33^^,^^37^^,^^40^^,^^42^^,^^43^^,^^52^^,^^63^^,^^65^^,^^73^^,^^75–77^^,^^99^^,^^100^^,^^108^^,^^119^^,^^122^^,^^124–128^^,^^134^

**Associations by overall instrument scores.** Twenty-three studies provided quantitative data on the relationship between overall instrument scores for functional capacity and real-world functioning (Supplementary Table 9).^30^^,^^37^^,^^40^^,^^43^^,^^44^^,^^52^^,^^63^^,^^65^^,^^73^^,^^75–77^^,^^81^^,^^100^^,^^108^^,^^119^^,^^123^^,^^124^^,^^126–128^^,^^130^^,^^133^ Thirteen of the 23 studies showed a significant positive association between greater functional capacity and greater real-world functioning based on at least 1 set of instrument scores.^40^^,^^52^^,^^63^^,^^73^^,^^75^^,^^77^^,^^108^^,^^119^^,^^123^^,^^124^^,^^128^^,^^130^^,^^133^ The remaining 10 studies either showed no significant association,^37^^,^^65^^,^^76^^,^^81^^,^^100^^,^^126^^,^^127^ or did not report sufficient data to establish significance.^30^^,^^43^^,^^44^ Relevant effect size data on the association between at least 1 set of instruments for functional capacity and real-world functioning were reported by 18 studies (Table 4).^30^^,^^37^^,^^40^^,^^43^^,^^52^^,^^63^^,^^65^^,^^73^^,^^75–77^^,^^100^^,^^108^^,^^119^^,^^124^^,^^126–128^ Of these, 4 found a large association,^52^^,^^73^^,^^119^^,^^128^ 7 a medium association,^40^^,^^43^^,^^73^^,^^75^^,^^77^^,^^108^^,^^124^ 11 a small association^30^^,^^37^^,^^43^^,^^52^^,^^63^^,^^65^^,^^76^^,^^77^^,^^100^^,^^126^^,^^127^; no study found a minimal association. Associations were generally consistent between the different instrument sets used within each study reporting relationship data for at least 2 instrument sets with overall scores.^30^^,^^43^^,^^44^^,^^52^^,^^63^^,^^73^ However, for Ventura et al.,^77^ small associations were found between real-world functioning measured using the SFS and functional capacity, which were significant when functional capacity was measured using the CAI, but not the SCoRS.

**Associations by subtest or domain scores.** Sixteen studies provided quantitative data on the relationship between functional capacity and real-world functioning exclusively in terms of subtest or domain-specific scores (Supplementary Table 9).^33^^,^^41^^,^^42^^,^^46^^,^^58^^,^^61^^,^^99^^,^^118^^,^^120–122^^,^^125^^,^^129^^,^^131^^,^^132^^,^^134^ Eleven studies reported subtest or subscale scores for the SLOF (*n* = 10)^41^^,^^42^^,^^46^^,^^61^^,^^99^^,^^118^^,^^120–122^^,^^125^ and SFS (*n* = 1).^134^ Mixed findings were reported for all except Giordano et al.,^42^ who found a significant positive association between functional capacity measured using the CAI and real-world everyday life skills, interpersonal relationships and work skills measured using the SLOF. For the 1 study using the SFS, there was a significant positive association between greater performance in all 7 real-world SFS domains assessed (withdrawal, interpersonal relations, social participation, recreation, self-reliance, and execution and employment) and greater functional capacity measured using the SCoRS.^134^ Relevant effect size data on the association between at least 1 set of instrument tests/subtests for functional capacity and real-world functioning were reported by 5 studies (Supplementary Table 9; Supplementary Table 10).^42^^,^^99^^,^^122^^,^^125^^,^^134^ Of these, 4 found a large association,^42^^,^^122^^,^^125^^,^^134^ 5 found a medium association,^42^^,^^99^^,^^122^^,^^125^^,^^134^ 2 found a small association,^99^^,^^122^ and 1 found a minimal association.^122^

Five studies provided relationship data based on real-world milestone scores for residential status (*n* = 4),^33^^,^^58^^,^^129^^,^^131^ employment (*n* = 1),^132^ education (*n* = 1),^129^ marital status (*n* = 1),^129^ social contacts (*n* = 1),^129^ and financial independence (*n* = 1)^33^ (Supplementary Table 9). One reported effect size data and found a small difference in residential and financial independence by functional capacity measured with both the UPSA-B and SSPA, which was significant for all associations except between residential independence and the UPSA-B (Supplementary Table 9; Supplementary Table 10).^33^ Three reported no significant association between functional capacity and real-world milestones (residential status^58^^,^^131^ and employment^132^). Mixed findings were reported by Olsson et al.,^129^ where a significant association was reported for greater functional capacity with greater odds of higher education and lower functional capacity with greater odds of supported housing. However, no significant association was found between functional capacity and marital status or social contacts.^129^

#### Interventional Studies

Of the 16 interventional studies, 2 (12.5%) provided quantitative data, including relevant effect size data, on the relationship between total scores for functional capacity and subscale scores for real-world functioning, and found mixed results (Supplementary Table 9).^135^^,^^136^ Gupta et al.^135^ found a medium significant positive association between the UPSA and SLOF activities and between the SSPA and SLOF interpersonal relationships, with all other associations small but non-significant (UPSA and SLOF interpersonal relationships, UPSA and SLOF work skills; SSPA and SLOF activities, SSPA and SLOF work skills; Medication Management Ability Assessment (MMAA) and SLOF interpersonal relationships, MMAA and SLOF activities, MMAA and SLOF work skills).^135^ For Twamley et al.^136^ a medium significant association was found between the UPSA and employment attainment for the intervention group who received individual placement and support, but a minimal non-significant association was found for the control group who received conventional vocational rehabilitation.^136^ No study reported longitudinal data on the relationship.

In summary, a clear and consistent pattern emerged across studies included in the review. Cognitive performance showed robust, typically medium-to-large associations with functional capacity across a range of instruments and study designs. In contrast, associations between functional capacity and real-world functioning were smaller, more variable, and frequently statistically non-significant. Similarly, relationships between functional capacity and real-world outcomes were inconsistent, especially when assessed using milestone-based indicators such as employment or residence independence. These findings suggest a hierarchical pattern in which cognitive performance is more proximally linked to performance-based functional skills, whereas real-world functioning reflects a more distal, multifactorial outcome influenced by non-cognitive factors including symptoms, motivation, and environmental context.

## Discussion

To our current knowledge, this is the largest systematic review to assess the relationship between cognitive performance and functioning in schizophrenia by synthesizing evidence from 104 primary research articles. This systematic literature review confirms that the MCCB, UPSA, and SLOF were the most frequently used measures for assessing cognitive performance, functional capacity, and real-world functioning, respectively. Additionally, a robust relationship between cognitive performance and functional capacity was demonstrated across measures, highlighting the central role of cognition in the performance of everyday tasks under simulated conditions. However, cognitive performance and functional capacity demonstrated more variable and attenuated associations by subtest or domain scores and with real-world functioning, suggesting that additional factors mediate these relationships.

The identification of a generally significant, medium-to-large positive association between cognitive performance and functional capacity in individuals with schizophrenia across most relevant studies, both observational and interventional, is consistent with previous research.^21–24^ This trend was strongest between the MCCB and UPSA, and was demonstrated longitudinally, with changes in cognitive performance showing a mostly significant, positive association with changes in functional capacity over time. Although relatively few studies reported detailed effect size data for a longitudinal relationship, those that did found a medium-to-large association. Collectively, this evidence reinforces the consensus that cognitive performance appears to be a key contributor to the ability to perform everyday functional skills in schizophrenia and that functional capacity is a proximal cognitive surrogate.^30^^,^^37^^,^^139^^,^^140^ This correlation is consistent with FDA-National Institute of Mental Health-MATRICS guidelines that clinical trials for cognitive-enhancing drugs in schizophrenia include “co-primary” standardized measures for cognitive and functional performance.^29^ However, this recommendation may be compromised by the fact that multiple studies have shown that improving cognitive performance without a psychosocial intervention is associated with reduced benefits on real-world functioning.^141^

The relationship between cognitive performance and real-world functioning was typically smaller and more variable than with functional capacity across both observational and interventional studies. In addition, the strength and consistency of relationships between cognitive performance and functional capacity and between cognitive performance and real-world functioning were more variable across subtest scores and individual domains, revealing important nuances in the strength of association. Although this highlights the importance of accounting for potential confounding factors, including negative symptoms and psychosocial factors, such as self-efficacy, interpersonal relationships, or socioeconomic status, it also suggests that heterogeneity in real-world functioning measures likely contributed to differences in the strength and consistency of observed associations. While structured psychometric instruments, such as rating scales or interviewer-based assessments, generally yielded more consistent relationships, milestone-based outcomes (eg, employment, residential independence) showed more variable findings. Though real-world milestones are helpful indicators of socioeconomic factors, they have limited time-sensitivity and are difficult to standardize, which may have contributed to the availability of effect size data in this review.^16^ In alignment with our mixed findings, Kharawala et al.^22^ found a smaller significant association between real-world functioning and cognitive performance than with functional capacity. This small association may be partially attributable to the substantial proportion of studies reporting domain-specific scores or real-world milestones rather than overall instrument scores.

When examining the relationship between cognitive performance and real-world milestones, most studies reported a significant association, which was small-to-medium in the few studies reporting relevant effect size data. Similarly to Kharawala et al.,^22^ living status and employment were the real-world functioning elements most shown to be significantly associated with cognitive performance, where poorer employment status and living status were positively associated with poorer cognitive performance. Together with the estimated unemployment rates of 80%–90% among individuals with schizophrenia,^142–144^ there is a clear unmet need for integrated treatment strategies for schizophrenia that improve clinical symptoms and translate to meaningful functional outcomes, including employment. While some interventions such as cognitive remediation, psychosocial treatments, and aerobic exercise have demonstrated modest but meaningful effects on cognitive outcomes, their uptake in routine care remains limited.^141^^,^^145–149^ Contributing factors may include service-level resource constraints, a lack of clinician training or awareness of available cognitive interventions, limited reimbursement for cognitive training and the under-recognition of functional outcomes as treatment priorities. Bridging this gap requires not only evidence of efficacy for these treatment strategies, but also implementation-focused research to address real-world barriers. Furthermore, despite the central role of cognitive performance in shaping functional potential in schizophrenia, cognitive screening is not routinely implemented in clinical practice, highlighting a persistent research-to-practice gap.^150^ While the UPSA/UPSA-B, the most used psychometric instrument to assess functional capacity in this review, assess functional capacity and elements of cognition indirectly, it does not directly assess core cognitive domains, such as memory and executive functioning.^140^^,^^151^ Therefore, the development of assessments that are both feasible in routine clinical practice and able to examine cognitive performance and functional capacity simultaneously, would enable more efficient detection and monitoring of cognitive symptoms and related aspects of functioning.

Most studies included in this review demonstrated a small-to-large, positive association between greater functional capacity and greater real-world functioning. However, all interventional studies and a subset of observational studies assessing the relationship reported mixed or non-significant findings, or lacked sufficient data to establish strength or significance. This variability highlights the complexity of mapping functional capacity to everyday outcomes, suggesting that while performance-based measures of functioning capture an important aspect of functioning, they do not fully reflect the lived realities of individuals with schizophrenia. Therefore, functional capacity may be best understood as an intermediate factor between cognition and real-world functioning, moderated by broader psychosocial and environmental factors. For example, the relationship between functional capacity and real-world functioning may differ across socioeconomic contexts. In low- and middle-income settings, where access to supported employment, community mental health services, and social safety nets may be more limited, the gap may be wider between what individuals are capable of performing under controlled conditions and what they are able to achieve in everyday life.^19^^,^^101^ Our findings are mostly consistent with Szabo et al.^152^ who examined the relationship between the UPSA and real–world functioning measures, predominantly the SLOF, revealing a borderline-significant correlation. These observations somewhat contrast with the consensus in Kharawala et al.^22^ in which functional capacity is understood to bridge the relationship between cognitive performance and functioning. However, Kharawala et al.^22^ acknowledge that functioning in schizophrenia is primarily moderated by other factors, including self-efficacy and negative symptoms, with the latter suggested to have a greater impact on functioning than other symptoms in individuals.^153^ This suggests that functional outcomes cannot be fully accounted for by cognitive or performance-based measures of functional capacity alone. Therefore, meaningful improvements in these domains require recovery-focused strategies that combine the treatment of clinical symptoms with person-centered psychosocial support, in line with the ICF model’s emphasis on addressing contextual factors to enhance real-world functioning.^5^

Further complicating interpretation of the relationship between cognitive performance, functional capacity, and real-world functioning in existing literature is the frequent conceptual and methodological conflation between functional capacity and real-world functioning measures.^23^^,^^24^^,^^27^ This limited distinction may distort estimates of the relationship between cognitive performance and functioning, potentially overstating the impact of cognitive-targeted interventions on real-world outcomes. Given that functional impairment is a core component of schizophrenia in the Diagnostic and Statistical Manual of Mental Disorders, Fifth Edition and International Classification of Diseases, 11th Revision,^12^^,^^154^ this conflation may also influence the assessment of individuals’ functional status, treatment, and recovery in clinical practice.

This systematic literature review has several strengths. It is the first to examine the 3-factor relationship between cognitive performance, functional capacity, and real-world functioning and adhere to a fully systematic methodology. Additionally, stringent inclusion criteria were employed to ensure greater methodological quality among included studies, such as a minimum sample size of individuals with schizophrenia and pre-identified standardized psychometric instruments to measure the outcome constructs of interest. The inclusion of studies based on the instruments employed helped to mitigate the lack of distinction between functional capacity and real-world functioning often seen in existing literature. Furthermore, the MCCB, UPSA, and SLOF, the most used instruments among included studies, are widely considered the leading instruments for their respective domains.^17^^,^^30^^,^^133^

However, this review is also subject to limitations. Firstly, most included studies were cross-sectional, which limited the ability to establish temporal associations. Secondly, comparability across studies was limited by heterogeneity in real–world outcome measures, ranging from clinician ratings to objective milestones, with clinician-rated or performance-based outcomes typically reporting stronger associations.^22^ Thirdly, the diversity of outcome types, psychometric tools, and reported metrics, including the absence of a standardized effect size, precluded the possibility of conducting a meaningful meta-analysis within the scope of this review. However, future research may wish to conduct an exploratory post hoc quantitative synthesis among subsets of studies with sufficiently comparable data. Similarly, a formal quality assessment was not conducted due to the wide range of study designs, outcome measures, and reporting methods, which could not be captured by a single standardized risk-of-bias assessment tool. To circumvent this, a minimum methodological standard was ensured through the application of stringent eligibility criteria, including minimum sample size thresholds, the requirement for validated psychometric instruments, and the exclusion of case reports, conference abstracts, and articles lacking sufficient methodological detail. Nevertheless, the absence of a formal risk-of-bias assessment tool furthers methodological considerations such as the potential for publication bias where non-significant findings are unpublished, and selection bias introduced in interventional studies. For example, given that interventional studies typically employ more stringent inclusion criteria, require informed consent, and are more demanding than observational studies in terms of the commitment and completion of trial visits and assessments, enrolled participants may have greater baseline cognitive performance and functioning than the broader patient population. Furthermore, the stringent nature of the inclusion criteria in interventional studies may have resulted in smaller magnitudes of association with fewer significant associations between cognitive performance, functional capacity, and real-world functioning relative to observational studies. Therefore, the relationships between cognitive performance, functional capacity, and real-world functioning captured in this systematic review may not be generalizable to more diverse or clinically representative populations with schizophrenia.

Another key methodological limitation is that the search strategy may not have retrieved all relevant articles due to the exclusion of grey literature, non-English language publications, and studies using psychometric instruments not listed in Table 1. A further limitation is the geographic distribution of included studies, with the majority conducted in high-income countries (the United States, Japan, and Italy). This concentration limits the generalizability of findings to low- and middle-income contexts, where social support systems, healthcare infrastructure, and local economic opportunities may substantially alter the relationship between functional capacity and real-world outcomes. Moreover, adapted instruments and high-quality data from low- and middle-income settings remain limited, further constraining cross-context comparisons*.*^19^^,^^101^ Future research should explore whether differences in patient demographics and clinical characteristics affect the relationship between cognitive performance and functioning in schizophrenia.

## Conclusion

The findings of this systematic review are largely consistent with previous research suggesting a moderate-to-strong relationship between cognitive performance and functional capacity in schizophrenia, contrasted with more variable associations between cognitive performance and real-world functioning, and functional capacity and real-world functioning. These findings support the conceptualization of functional capacity as an intermediate construct linking cognitive abilities to everyday outcomes. Although this review highlights the central role of cognitive performance in shaping functional abilities, the translation of cognitive gains into meaningful real-world improvements remains complex, likely moderated by a range of clinical, psychosocial, and environmental factors.

This review necessitates the adoption of a multimodal, person-centered approach to outcome evaluation in schizophrenia that integrates cognitive, functional, and contextual domains. Future research should prioritize longitudinal and interventional designs, improved standardization of outcome measures, and exploration of moderators that could influence the relationship between capacity and real-world functioning. Such endeavor will be critical for optimizing treatment strategies and improving meaningful functional outcomes in this population.

## Supplementary Material

Cog_and_func_SLR_revised_suppl_clean_sbag121

## Data Availability

As a systematic literature review article, a data sharing statement is not applicable. The protocol can be found on the PROSPERO register: https://www.crd.york.ac.uk/PROSPERO/view/CRD42024532729.
