# Supplementary material for: Exploring the Relationship between Cognitive Performance, Performance-Based Functioning, and Everyday Functioning in People with Schizophrenia: a Systematic Literature Review
Source: Schizophr Bull. 2026 Aug 20;52(5):sbag121. doi: 10.1093/schbul/sbag121 (PMC13492084; doi:10.1093/schbul/sbag121)
Supplement: Supplementary file 1 [file cog_and_func_slr_revised_suppl_clean_sbag121.docx]

# Supplementary material

## Methods

### Search strategy

#### Supplementary Table 1. Literature database searches and number of hits for MEDLINE and Embase

| No. | Search strategy for MEDLINE/Embase via Embase.com | Number of hits |
| --- | --- | --- |
| #1 | 'schizophrenia spectrum disorder'/exp/mj OR 'early psychosis'/exp/mj OR 'first episode psychosis':ti,ab,kw | 155275 |
| #2 | 'matrics consensus cognitive battery' OR mccb:ti,ab,kw OR cogstate:ti,ab,kw OR 'cambridge neuropsychological test automated battery' OR 'cantab':ti,ab,kw OR 'brief assessment of cognition in schizophrenia' OR bacs:ti,ab,kw OR 'bac-app':ti,ab,kw OR drs:ti,ab,kw OR 'drs-2':ti,ab,kw OR mdrs:ti,ab,kw OR 'mattis dementia rating scale' OR 'brief battery of the social cognition psychometric evaluation' OR 'bb-scope':ti,ab,kw OR 'cogstate schizophrenia battery' OR CSB:ti,ab,kw OR ‘repeatable battery for the assessment of neuropsychological status’ OR RBANS:ti,ab,kw | 23580 |
| #3 | 'cognition'/exp/mj | 1099273 |
| #4 | #2 OR #3 | 1119777 |
| #5 | 'ucsd performance-based skills assessment' OR upsa*:ti,ab,kw OR 'schizophrenia cognition rating scale' OR 'scors':ti,ab,kw OR 'virtual reality functional capacity assessment tool' OR vrfcat OR 'social skills performance assessment' OR 'sspa':ti,ab,kw OR 'maryland assessment of social competence' OR 'masc':ti,ab,kw OR 'cognitive assessment interview' OR 'cai':ti,ab,kw OR 'functional skills assessment and training' OR 'funsat':ti,ab,kw OR 'medication management ability assessment' OR MMAA:ti,ab,kw | 10503 |
| #6 | 'functional assessment'/exp OR 'social competence'/exp | 81499 |
| #7 | 'functional capacity':ti,ab,kw OR 'functional competence':ti,ab,kw OR 'social competence':ti,ab,kw | 31652 |
| #8 | #5 OR #6 OR #7 | 120249 |
| #9 | 'specific levels of functioning scale' OR 'slof':ti,ab,kw OR 'quality of life scale' OR qls:ti,ab,kw OR 'social functioning scale' OR sfs:ti,ab,kw OR 'social adjustment scale' OR 'sas-m':ti,ab,kw OR (sas:ti,ab,kw AND function*:ti,ab,kw) OR ‘world health organization disability assessment schedule’ OR WHODAS:ti,ab,kw | 27432 |
| #10 | employment:ti,ab,kw OR unemployment:ti,ab,kw OR marriage:ti,ab,kw OR 'homeless*':ti,ab,kw OR 'stable relationship*' OR 'college attendance':ti,ab,kw OR 'college graduation':ti,ab,kw OR 'university attendance':ti,ab,kw OR 'university graduation':ti,ab,kw | 161028 |
| #11 | 'employment status'/exp | 50641 |
| #12 | 'real-world functioning':ti,ab,kw OR 'social functioning':ti,ab,kw OR 'work skills':ti,ab,kw OR vocational:ti,ab,kw OR 'functional milestones':ti,ab,kw OR 'functional outcome*':ti,ab,kw OR 'functional disability':ti,ab,kw | 141086 |
| #13 | #9 OR #10 OR #11 OR #12 | 346379 |
| #14 | #4 AND #8 | 7737 |
| #15 | #8 AND #13 | 13490 |
| #16 | #4 AND #13 | 18928 |
| #17 | #14 OR #15 OR #16 | 38331 |
| #18 | #1 AND #17 | 3066 |
| #19 | #18 AND ('Chapter'/it OR 'Conference Abstract'/it OR 'Conference Paper'/it OR 'Editorial'/it OR 'Letter'/it OR 'Note'/it OR 'Tombstone'/it) | 1046 |
| #20 | #18 NOT ('chapter'/it OR 'conference abstract'/it OR 'conference paper'/it OR 'editorial'/it OR 'letter'/it OR 'note'/it OR 'tombstone'/it) | 2020 |
| #21 | #18 NOT ('chapter'/it OR 'conference abstract'/it OR 'conference paper'/it OR 'editorial'/it OR 'letter'/it OR 'note'/it OR 'tombstone'/it) AND [english]/lim | 1,926 |
|  | After deduplication within search results for database | 1,906 |

#### Supplementary Table 2. Literature database searches and number of hits for Cochrane Database of Systematic Reviews

| No. | Search strategy for Cochrane Database of Systematic Reviews via Cochrane Library | Number of hits |
| --- | --- | --- |
| #1 | MeSH descriptor: [Schizophrenia Spectrum and Other Psychotic Disorders] explode all trees | 12240 |
| #2 | "early psychosis" OR "first episode psychosis" | 1208 |
| #3 | #1 OR #2 | 12811 |
| #4 | "matrics consensus cognitive battery" OR mccb:ti,ab,kw OR cogstate:ti,ab,kw OR "cambridge neuropsychological test automated battery" OR "cantab":ti,ab,kw OR "brief assessment of cognition in schizophrenia" OR "bacs":ti,ab,kw OR "bac-app":ti,ab,kw OR drs:ti,ab,kw OR "drs-2":ti,ab,kw OR mdrs:ti,ab,kw OR "mattis dementia rating scale" OR "brief battery of the social cognition psychometric evaluation" OR "bb-scope":ti,ab,kw OR “cogstate schizophrenia battery” OR CSB:ti,ab,kw OR “repeatable battery for the assessment of neuropsychological status” OR RBANS:ti,ab,kw | 3069 |
| #5 | MeSH descriptor: [Cognition] explode all trees | 16189 |
| #6 | #4 OR #5 | 18975 |
| #7 | "ucsd performance-based skills assessment" OR upsa*:ti,ab,kw OR "schizophrenia cognition rating scale" OR "scors":ti,ab,kw OR "virtual reality functional capacity assessment tool" OR vrfcat OR "social skills performance assessment" OR sspa:ti,ab,kw OR "maryland assessment of social competence" OR masc:ti,ab,kw OR "cognitive assessment interview" OR cai:ti,ab,kw OR "functional skills assessment and training" OR funsat:ti,ab,kw OR “medication management ability assessment” OR MMAA:ti,ab,kw | 1061 |
| #8 | MeSH descriptor: [Functional Status] explode all trees | 152 |
| #9 | MeSH descriptor: [Social Skills] explode all trees | 421 |
| #10 | "functional capacity":ti,ab,kw OR "functional competence":ti,ab,kw OR "social competence":ti,ab,kw | 6418 |
| #11 | #7 OR #8 OR #9 OR #10 | 7780 |
| #12 | "specific levels of functioning scale" OR slof:ti,ab,kw OR "quality of life scale" OR qls:ti,ab,kw OR "social functioning scale" OR sfs:ti,ab,kw OR "social adjustment scale" OR "sas-m":ti,ab,kw OR (sas:ti,ab,kw AND function*:ti,ab,kw) OR “world health organization disability assessment schedule” OR WHODAS:ti,ab,kw | 5034 |
| #13 | employment:ti,ab,kw OR unemployment:ti,ab,kw OR marriage:ti,ab,kw OR homeless*:ti,ab,kw OR “stable relationship” OR “stable relationships” OR “college attendance”:ti,ab,kw OR “college graduation”:ti,ab,kw OR “university attendance”:ti,ab,kw OR “university graduation”:ti,ab,kw | 11241 |
| #14 | MeSH descriptor: [Employment] explode all trees | 2831 |
| #15 | "real-world functioning":ti,ab,kw OR "social functioning":ti,ab,kw OR "work skills":ti,ab,kw OR vocational:ti,ab,kw OR (functional NEXT milestone*):ti,ab,kw OR (functional NEXT outcome*):ti,ab,kw OR "functional disability":ti,ab,kw | 19082 |
| #16 | #12 OR #13 OR #14 OR #15 | 35203 |
| #17 | #6 AND #11 | 294 |
| #18 | #11 AND #16 | 672 |
| #19 | #6 AND #16 | 781 |
| #20 | #17 OR #18 OR #19 | 1563 |
| #21 | #3 AND #20 | 325 |
| #22 | Cochrane Reviews | 19 |

#### Supplementary Table 3. Literature database searches and number of hits for PsycINFO

| No. | Search strategy for PsychInfo via APA | Number of hits |
| --- | --- | --- |
| #1 | **Index Terms**: {Schizophrenia} | 101,786 |
| #2 | **Keywords**: “early psychosis” OR **Keywords**: “first episode psychosis” OR **Abstract**: “early psychosis” OR **Abstract**: “first episode psychosis” | 5441 |
| #3 | #1 OR #2 | 105,522 |
| #4 | **Keywords**: “matrics consensus cognitive battery” OR mccb OR cogstate OR “cambridge neuropsychological test automated battery” OR cantab OR “brief assessment of cognition in schizophrenia” OR bacs OR “bac-app” OR drs OR “drs-2” OR mdrs OR “mattis dementia rating scale” OR “brief battery of the social cognition psychometric evaluation” OR “bb-scope” OR “cogstate schizophrenia battery” OR CSB OR “repeatable battery for the assessment of neuropsychological status” OR RBANS  *OR* **Abstract**: “matrics consensus cognitive battery” OR mccb OR cogstate OR “cambridge neuropsychological test automated battery” OR cantab OR “brief assessment of cognition in schizophrenia” OR bacs OR ‘bac-app’ OR drs OR “drs-2” OR mdrs OR “mattis dementia rating scale” OR “brief battery of the social cognition psychometric evaluation” OR “bb-scope” OR “cogstate schizophrenia battery” OR CSB OR “repeatable battery for the assessment of neuropsychological status” OR RBANS | 6,134 |
| #5 | **Index Terms**: {Cognition} | 55,249 |
| #6 | #4 OR #5 | 69,546 |
| #7 | **Keywords**: “ucsd performance-based skills assessment” OR upsa* OR “schizophrenia cognition rating scale” OR scors OR “virtual reality functional capacity assessment tool” OR vrfcat OR “social skills performance assessment” OR sspa OR “maryland assessment of social competence” OR masc OR “cognitive assessment interview” OR cai OR “functional skills assessment and training” OR funsat OR “medication management ability assessment” OR MMAA  *OR* **Abstract**: “ucsd performance-based skills assessment” OR upsa* OR “schizophrenia cognition rating scale” OR scors OR “virtual reality functional capacity assessment tool” OR vrfcat OR “social skills performance assessment” OR sspa OR “maryland assessment of social competence” OR masc OR “cognitive assessment interview” OR cai OR “functional skills assessment and training” OR funsat OR “medication management ability assessment” OR MMAA | 2030 |
| #8 | **Index Terms**: {Functional Status} *OR* **Index Terms**: {Social Skills} | 25,428 |
| #9 | **Keywords**: “functional capacity” OR “functional competence” OR “social competence” *OR* **Abstract**: “functional capacity” OR “functional competence” OR “social competence” | 10,610 |
| #10 | #7 OR #8 OR #9 | 33,093 |
| #11 | **Keywords**: “specific levels of functioning scale” OR slof OR “quality of life scale” OR qls OR “social functioning scale” OR sfs OR “social adjustment scale” OR “sas-m” OR (sas AND function*) OR “world health organization disability assessment schedule” OR WHODAS  *OR* **Abstract**: “specific levels of functioning scale” OR slof OR “quality of life scale” OR qls OR “social functioning scale” OR sfs OR “social adjustment scale” OR “sas-m” OR (sas AND function*) OR “world health organization disability assessment schedule” OR WHODAS | 4162 |
| #12 | **Keywords**: employment OR unemployment OR marriage OR homeless* OR “stable relationship*” OR “college attendance” OR “college graduation” OR “'university attendance” OR “university graduation”  OR **Abstract**: employment OR unemployment OR marriage OR homeless* OR “stable relationship*” OR “college attendance” OR “college graduation” OR “'university attendance” OR “university graduation” | 133,471 |
| #13 | **Index Terms**: {Employment Status} | 20,673 |
| #14 | **Keywords**: “real-world functioning” OR “social functioning” OR “work skills” OR vocational OR “functional milestone*” OR “functional outcome*” OR “functional disability”  *OR* **Abstract**: “real-world functioning” OR “social functioning” OR “work skills” OR vocational OR “functional milestone*” OR “functional outcome*” OR “functional disability” | 66,485 |
| #15 | #11 OR #12 OR #13 OR #14 | 195,322 |
| #16 | #6 AND #10 (Cognition + Function) | 1,146 |
| #17 | #10 AND #15 (Function + RWF) | 3,289 |
| #18 | #6 AND #15 (Cognition + RWF) | 2,018 |
| #19 | #16 OR #17 OR #18 | 5,931 |
| #20 | #3 AND #19 | 1,370 |
|  | Limits: Journal | 1,201 |
|  | After deduplication within search results for database | 1,196 |

### Data synthesis

#### Statistical analysis methods for unpublished data from Miller et al.^33^

1. **Examining the relationship between cognitive performance and functional capacity (n=480)**

Pearson’s correlation coefficients were calculated between MCCB subtest scores (composite score of the Trail Making Test Part A, Maryland Letter-Number Span and Animal Fluency [Total number named out of 48]) and UPSA-B and SSPA scores

2. **Examining the relationship between cognitive performance and real-world functioning (n=480)**

A t-test was performed to assess group differences in residential independence and financial independence by MCCB subtest scores

3. **Examining the relationship between functional capacity and real-world functioning (n=480)**

A t-test was performed to assess group differences in residential independence and financial independence by UPSA-B and SSPA scores

#### Supplementary Table 4. Overlapping datasets identified across all articles

| **Cohort/dataset** | **Included publications** | **Excluded overlapping publications** |
| --- | --- | --- |
| **Epidemiology-Genetics (Epigen) Program in Psychiatry at the Johns Hopkins School of Medicine, US** | Bowie et al. 2010^120^ | Mausbach et al. 2011^153^ |
|  |  | Mausbach et al. 2010^154^ |
| **SCOPE 3 (Miami and Dallas), US** | Pinkham et al. 2016^36^  Miller et al. 2021^33^* | Robertson et al. 2014^155^ |
|  |  | Kalin et al. 2015^156^ |
|  |  | Strassnig et al. 2015^157^ |
| **SCOPE 5 (Miami, Dallas, North Carolina), US** | Pinkham et al. 2018^67^  Miller et al. 2021^33^* | Deste et al. 2020^158^ |
|  |  | Fundora et al. 2021^159^ |
| **VALERO 2 (Miami, Atlanta, San Diego), US** | Gould et al. 2015^99^ | Durrand et al. 2015^160^ |
|  |  | Strassnig et al. 2015^157^ |
| **VALERO 1 (Atlanta and San Diego), US** | Harvey et al. 2011^133^  Burton et al. 2013^84^* | Harvey et al. 2013^161^ |
|  |  | Sabbag et al. 2012^162^ |
|  |  | Strassnig et al. 2015 ^157^ |
| **VRFCAT validation study dataset (South Carolina, Miami, California), US** | Keefe et al. 2016^52^ | Harvey et al. 2020^163^ |
|  |  | Harvey et al. 2019^164^ |
|  |  | Ruse et al. 2014^165^ |
| **Dataset of older outpatients with schizophrenia enrolled in a longitudinal study in New York, US** | Bowie et al. 2008^121^ | Bowie et al. 2006^166^ |
|  |  | Gould et al. 2012^167^ |
|  |  | Strassnig et al. 2015^157^ |
| **Patients recently hospitalized for an acute exacerbation of psychotic symptoms from centers in the US, Russia, India, Ukraine, Romania, and Colombia** | Harvey et al. 2013*^87^* | Harvey et al. 2015^168^ |
| **Research participants at the**  **University of California San Diego Advanced Center for Interventions and Services Research, US** | Mausbach et al. 2008^58^ | Mausbach et al. 2007^149^ |
| **Outpatients across 26 university psychiatric clinics and/or mental health departments in Italy** | Galderisi et al. 2016^41^ | Galderisi et al. 2018^169^ |
| **Outpatients or partial hospital patients from 2 community mental**  **health centers at University of Medicine and Dentistry of New Jersey, US; New York Presbyterian Hospital–Weill Medical College of Cornell University–Westchester Division, US; Zucker-Hillside Hospital, US and Thresholds, Inc, US** | Silverstein et al. 2010^70^ | Silverstein et al. 2011^170^ |
| **Patients from outpatient treatment**  **clinics at Nasukougen Hospital, Japan** | Okada et al. 2021^134^ | Okada et al. 2021^171^ |
| **Inpatients at rehabilitation center or inpatient treatment facility at John Umstead Hospital, US** | Keefe et al. 2006^54^ | Keefe et al. 2006^138^ |

*Unique analysis.

SCOPE, Social Cognition Psychometric Evaluation; VALERO, Validation of Everyday Real-World Outcomes; US, United States; VRFCAT, Virtual Reality Functional Capacity Assessment Tool.

## Results

### *Supplementary Table 5. Characteristics and key results of final included studies examining the relationship between cognitive performance and functional capacity (n=63)*

| **Author** | **Publication Year** | **Country (stated or clear from authorship)** | **Study design** | **Sample size^a^** | **Instrument pair(s)** | **Subtest/**  **domain score (overall/total score not reported)** | **Key results** | **Interpretation of association data** | | |
| --- | --- | --- | --- | --- | --- | --- | --- | --- | --- | --- |
|  |  |  |  |  |  |  |  | **Significance of association** | **Effect size** | |
|  |  |  |  |  |  |  |  |  | **N, analysis** | **Strength of association** |
| **Observational studies** | | | | | | | | | | |
| Bechi et al.^37^ | 2017 | Italy | Cross-sectional | 79 | BACS and UPSA |  | BACS total and UPSA-B total (r=0.415, p=0.001) | Greater NC performance showed a significant association with greater FC performance | 79 | Medium positive relationship between BACS and UPSA-B |
| Burton et al.^84^ | 2013 | US | Cross-sectional | 183 | MCCB and UPSA-B MCCB and SSPA |  | MCCB modified cognitive composite and UPSA-B total score (r=0.459, p<0.01) MCCB modified cognitive composite and SSPA mean score (r=0.146, p<0.05) | Greater NC performance showed a significant association with greater FC performance | 183 | Medium positive relationship between MCCB and UPSA-B; small positive relationship between MCCB and SSPA |
| Chia et al.^38^ | 2010 | Singapore | Longitudinal | 103 | BACS and SCoRS |  | SCoRS global rating and BACS (r_s_=-0.264, p=0.008) | Greater GC performance showed a significant association with greater FC performance | 103 | Small negative relationship between SCoRS global rating and BACS |
| Fervaha et al.^39^ | 2015 | US | Cross-sectional | 176 | MCCB and UPSA |  | MCCB and UPSA (r=0.57, p<0.001) | Greater NC performance showed a significant association with greater FC performance | 176 | Large positive relationship between MCCB and UPSA |
| Fujino et al.^40^ | 2016 | Japan | Cross-sectional | 93 | MCCB and UPSA-B |  | MCCB significantly predicted UPSA-B scores (r=0.709, p<0.01) | Greater GC performance showed a significant association with greater FC performance | 93 | Large positive relationship between MCCB and UPSA-B |
| Galderisi et al.^41^ | 2016 | Italy | Cross-sectional | 921 | MCCB and UPSA-B |  | NC MCCB showed significant direct effects on UPSA-B (β=0.502)  [significant relationship inferred as ns paths were trimmed] | Greater NC performance showed significant direct association with greater FC performance | Effect size not interpreted - only regression coefficient value reported | |
| Giordano et al.^42^ | 2022 | Italy | Cross-sectional | 580 | MCCB and UPSA-B MCCB and CAI |  | MCCB composite score and UPSA-B total score (r=0.569, p<0.0004) MCCB composite score and CAI composite global score (r=-0.500, p<0.0004) | Greater GC performance showed a significant association with greater FC performance | 580 | Large positive relationship between MCCB composite score and UPSA-B total score; large negative relationship between MCCB composite score and CAI composite global score |
| Green et al.^30^ | 2011 | US | Cross-sectional | 166 | MCCB and UPSA MCCB and CAI |  | MCCB and UPSA (r=0.67)  MCCB and UPSA short form (r=0.53)  MCCB and CAI (r=0.23) [p-values not reported] | Greater GC performance showed an association with greater FC performance | 163 | Large positive relationship between: MCCB and UPSA, MCCB and UPSA short form Small positive relationship between MCCB and CAI |
| Green et al.^43^ | 2008 | US | Cross-sectional | 176 | MCCB and UPSA MCCB and SCoRS MCCB and MASC |  | MCCB and MASC: conversational content (r=0.42), non-verbal content (r=0.32), effectiveness (r=0.40) MCCB and UPSA total score (r=0.61) MCCB and SCoRS, patient (r=-0.18), informant (r=-0.21), interviewer (r=-0.31) [p-values not reported] | Greater GC performance showed an association with greater FC performance | 156 | Medium positive relationship between MCCB and MASC conversational content, MASC non-verbal content, MASC effectiveness Large positive relationship between MCCB and UPSA total score Small negative relationship between MCCB and SCoRS/patient, SCoRS/informant; medium negative relationship between MCCB and SCoRS/interviewer |
| Hajdúk et al.^44^ | 2021 | US | Cross-sectional | 408 | MCCB and UPSA-B  MCCB and SSPA |  | MCCB, particularly working memory, showed a strong direct relationship with functional competence (UPSA) and social competence (SSPA) | Greater NC performance showed a direct association with FC | Effect size not interpreted - network analysis | |
| Harvey et al.^35^ | 2016 | US | Cross-sectional | 4310 | MCCB subset and UPSA-B |  | Cognition and functional capacity measures could be reduced to a single principal component explaining 50%–54% of the variance in CSP 572 (eigenvalues: 3.89 with adaptive function, 3.19 without) and 52% in FUNCAP (eigenvalue: 4.20 with adaptive function, 3.85 without) | Single-factor model shown as the best fit for GC and FC, indicating that they are not distinct factors and instead converge onto a single underlying dimension in schizophrenia | Effect size not interpreted - only % variance reported | |
| Higuchi et al.^45^ | 2017 | Japan | Cross-sectional | 294 | BACS (Japanese version) and SCoRS |  | BACS composite z-scores and SCoRS global rating, all patients (including at-risk-mental state and other disorders) (r=-0.362, p<0.0001), first-episode schizophrenia (r=-0.497, p=0.001), chronic schizophrenia (r=-0.373, p<0.0001) | Greater GC performance showed a significant association with greater FC | All patients, n=294; first-episode SCZ, n=38; chronic SCZ, n=135 | Medium negative relationship between BACS composite z-scores and SCoRS global rating (in all patients, first-episode schizophrenia subgroup, chronic schizophrenia subgroup) |
| Ho et al.^46^ | 2013 | US | Cross-sectional | 138 | RBANS and UPSA |  | RBANS significantly predicted performance in UPSA (B=0.766 ±0.089, p<0.01) | GC performance significantly predicted FC performance | Effect size not interpreted - only regression coefficient value reported | |
| Huang et al.^47^ | 2020 | Taiwan | Cross-sectional | 63 | BACS and UPSA-B | UPSA subscale | BACS and UPSA-B domains (patients with schizophrenia), Financial skill 1 (r=0.144, p=0.259), Financial skill 2 (0.425, p=0.001), Communication skill (r=0.429, p<0.001) | Greater GC performance showed a significant association with FC Financial skill 2 (paying a bill) and communication skills performance but showed a non-significant association with Financial skill 1 | 63 | Medium positive relationship between BACS and UPSA-B Financial skill 2 and communication domains Small positive relationship between BACS and UPSA-B Financial skill 1 domain |
| Hurford et al.^48^ | 2018 | US | Cross-sectional | 91 | MCCB and UPSA-B |  | MCCB and UPSA-B (r=0.58, p<0.0001) | Greater GC showed a significant association with greater FC performance | 91 | Large positive relationship between MCCB and UPSA |
| Jeon et al.^49^ | 2020 | Korea | Cross-sectional | 56 | MCCB and SCoRS |  | MCCB total score showed a strong significant relationship with SCoRS global score (r=-0.52, p<0.001) MCCB total score showed statistically significant correlations with 18 out of the 20 SCoRS items, and speed of processing was significantly correlated with all SCoRS items SCoRS global score showed statistically significant correlations with all 7 MCCB domains | Greater GC performance showed a significant association with greater FC | 56 | Large negative relationship between MCCB total score and SCoRS global score |
| Jeste et al.^50^ | 2003 | US | Cross-sectional | 110 | DRS and MMAA |  | DRS was a significant predictor of MMAA (B=0.383, p≤0.01) | Greater GC performance showed a significant association with greater FC | Effect size not interpreted - only regression coefficient value reported | |
| Kalache et al.^51^ | 2015 | Canada | Cross-sectional | 232 | MCCB and UPSA | UPSA subscale | GC was a strong predictor of UPSA, including Comprehension and Planning of Recreational Activities Skills (B=0.047 ±0.015, p=0.002), Financial Skills (B=0.095 ±0.019, p<0.001), Communication Skills (B=0.089 ±0.011, p<0.001), and Transportation Skills (B=0.064 ±0.018, p<0.001), but not Household Management Skills (B=-0.024 ±0.011, p=0.049) | Greater GC performance was a significant predictor of FC comprehension and planning of recreational activities, financial skills, communication skills and transportation skills but not household management | Effect size not interpreted - only regression coefficient value reported | |
| Keefe et al.^52^ | 2016 | US | Cross-sectional | 167 | MCCB and UPSA MCCB and VRFCAT |  | MCCB and UPSA-VIM (r=0.70, p<0.001) MCCB and VRFCAT, Total time (r=0.57, p<0.001), Total errors (r=0.39, p<0.001), Total progressions (r=0.45, p<0.001) | Greater GC showed a significant association with FC performance | 155 | Large positive relationship between MCCB and UPSA-VIM Large positive relationship between MCCB and VRFCAT, Total time Medium positive relationship between MCCB and VRFCAT, Total errors/VRFCAT, Total progressions |
| Keefe et al.^53^ | 2011 | US | Cross-sectional | 323 | MCCB and UPSA-B MCCB and CAI |  | MCCB and UPSA-B (r=0.60, p<0.001) MCCB and CAI (r=-0.14, p=0.066) | Greater GC showed a significant association with greater FC performance | 321 | Large positive relationship between MCCB and UPSA-B Small negative relationship between MCCB and CAI |
| Keefe et al.^54^ | 2006 | US | Cross-sectional | 60 | BACS and UPSA BACS and SCoRS |  | BACS composite score and UPSA (r=0.650, P<0.001) BACS and SCoRS: patient (r=-0.063, ns), informant (r=-0.415, p<0.01), interviewer (r=-0.540, p<0.001) | Greater GC showed a significant association with FC performance  [patient-rated SCoRS ns] | Between 55 and 59 | Large positive relationship between BACS composite score and UPSA Large negative relationship between BACS and SCoRS interviewer Medium negative relationship between BACS and SCoRS informant Minimal negative relationship between BACS and SCoRS patient |
| Keefe et al.^95^ | 2015 | US, Russia, Serbia, Ukraine | Cross-sectional | 395 | MCCB and SCoRS |  | MCCB at baseline and SCoRS total (r=-0.35, p<0.05) | Greater GC showed a significant association with FC performance | 230 | Medium negative relationship between MCCB at baseline and SCoRS total |
| Kumar et al.^55^ | 2016 | Canada | Cross-sectional | 60 | MCCB and UPSA |  | MCCB global score and UPSA total score (r=0.65, p<0.001) | Greater GC showed a significant association with greater FC performance | 60 | Large positive relationship between MCCB global score and UPSA total score |
| Ludwig et al.^56^ | 2017 | US | Cross-sectional | 38 | MCCB and UPSA-B MCCB and SSPA SCOPE and UPSA |  | MCCB composite and UPSA Total (r=0.489, p<0.01)  MCCB composite and SSPA average (r=0.384, p<0.05) SCOPE social cognitive tasks and UPSA Total: significant correlation (p<0.05) for ER-40, Eyes, Hinting, significant correlation (p<0.01) for RAD and TASIT  SCOPE social cognitive tasks and SSPA average: significant correlation (p<0.01) for ER-40, Hinting, significant correlation (p<0.05) for RAD | Greater NC performance showed a significant association with greater FC performance  Greater performance on 5/8 SC tests (emotional processing, theory of mind, social perception) showed a significant association with greater FC | 38 | Medium positive relationship between MCCB composite and UPSA Total Medium positive relationship between MCCB composite and SSPA average |
| Mahmood et al.^57^ | 2022 | US | Cross-sectional | 55 | MCCB and UPSA-B MCCB and SSPA |  | MCCB and UPSA (r=0.66, p<0.001) MCCB and SSPA (r=0.33, p=0.013) | Greater NC performance showed a significant association with greater FC performance | 55 | Large positive relationship between MCCB and UPSA Medium positive relationship between MCCB and SSPA |
| Masuzawa et al.^85^ | 2022 | Japan | Cross-sectional | 50 | BACS and SCoRS |  | Spearman's correlation between SCoRS-J global ratings and BACS-J composite scores:  - Patient's SCoRS rating r_s_=-0.152 [p-value not reported] - Informant's SCoRS rating r_s_=-0.496, p<0.008 - Interviewer's SCoRS rating r_s_=-0.504, p<0.008 | Greater GC performance showed a significant association with greater FC performance  [patient rating association, p-value not reported] | 50 | Large negative relationship between BACS-J composite scores and Interviewer's SCoRS global ratings Medium negative relationship between BACS-J composite scores and Informant's SCoRS global ratings Small negative relationship between BACS-J composite scores and patient's SCoRS global ratings |
| Mausbach, et al.^58^ | 2008 | US | Cross-sectional | 434 | DRS and UPSA |  | DRS was a significant predictor of UPSA score (t=10.23, p<0.001) | NC performance was a significant predictor of FC performance | Effect size not interpreted - only t value reported | |
| Mazhari et al.^59^ | 2017 | Iran | Cross-sectional | 35 | BACS and SCoRS |  | SCoRS global rating score and BACS z-score (r=-0.84, p<0.001) | Greater GC performance showed a significant association with greater FC performance | 35 | Large negative relationship between SCoRS global rating score and BACS z-score |
| Miller et al.^33^ | 2021 | US | Cross-sectional | 557 | MCCB and UPSA-B MCCB and SSPA |  | MCCB subtest and UPSA-B (r=0.40, p<0.001)^b^ MCCB subtest and SSPA (r=0.21, p<0.001)^b^ | Greater GC showed a significant association with FC performance | 480 | Medium positive relationship between MCCB subtest and UPSA-B Medium positive relationship between MCCB subtest and SSPA |
| Moore et al.^60^ | 2015 | US | Cross-sectional | 205 | RBANS and UPSA-B |  | In the final model, RBANS did not show a linear relationship with UPSA-B scores (t=-1.90, p=0.06. When RBANS z-scores were >−1 (low average–average performance), NC was not significantly related to UPSA-B scores | NC did not show a significant association with FC | Effect size not interpreted - only t value reported | |
| Mucci et al.^61^ | 2021 | Italy | Longitudinal | 618 | MCCB and UPSA-B |  | Higher baseline neurocognitive functioning (MCCB) was associated, as measured in the model by standardized regression coefficients, with positive change at 4-year follow-up in UPSA-B total score (β=1.138; 95% CI 0.807-1.469; p<0.001) | GC performance showed a significant positive association with FC | Effect size not interpreted - only regression coefficient value reported | |
| Muharib et al.^62^ | 2014 | Canada | Cross-sectional | 35 | MCCB and UPSA |  | Cognitively impaired patients performed significantly lower than normal-range patients (d=3.30, p<0.05) | FC performance was significantly different by GC performance | Cognitively impaired patients, n=21; normal-range cognition patients, n=14 | The difference between the cognitively impaired patients and the normal-range cognition patients is large |
| Nagendra et al.^63^ | 2020 | US | Cross-sectional | 169 | MCCB and UPSA  MCCB and SSPA |  | MCCB composite and UPSA-B (r=0.50, p<0.01) MCCB composite and SSPA (r=0.41, p<0.01) | NC performance showed a significant positive association with FC performance | 169 | Large positive relationship between MCCB composite and UPSA-B  Medium positive relationship between MCCB composite and SSPA |
| Olbert et al.^64^ | 2013 | US | Cross-sectional | 173 | MCCB and UPSA MCCB and MASC |  | MCCB composite score and UPSA total (r=0.69, p<0.01) MCCB composite score and MASC total (r=0.30, p<0.01) | Greater NC performance showed a significant association with greater GC performance | 173 | Large positive relationship between MCCB composite score and UPSA total Medium positive relationship between MCCB composite score and MASC total |
| Patterson et al.^148^ | 2001 | US | Cross-sectional | 83 | DRS and SSPA |  | SSPA total score and DRS (r=-0.56, p<0.01) | Greater GC showed a significant association with greater FC performance | 83 | Large negative relationship between SSPA total score and DRS |
| Pietrzak et al.^66^ | 2009 | US, Australia, and United Kingdom | Cross-sectional | 121 | MCCB and UPSA CogState and UPSA |  | UPSA total score and MCCB composite score (r=0.79, p<0.001) UPSA total score and CogState battery composite score (r=0.76, p<0.001) | Greater GC showed a significant association with greater FC performance | UPSA and MCCB, n=98; UPSA and CogState, n=93 | Large positive relationship between UPSA total score and MCCB composite score Large positive relationship between UPSA total score and CogState battery composite score |
| Pinkham et al.^36^ | 2016 | US | Cross-sectional | 179 | MCCB and UPSA-B MCCB and SSPA SCOPE and UPSA-B | MCCB subtest SCOPE subtest | Neurocognition (MCCB tests) and UPSA total: TMT-A (r=-0.270, p<0.001), Symbol coding (r=0.264, p<0.001), HVLT-R (r=0.421, p<0.001), LNS (r=0.544, p<0.001), Animal naming (r=0.174, p<0.05) Neurocognition (MCCB tests) and SSPA average: TMT-A (r=-0.103, ns), Symbol coding (r=0.301, p <0.001), HVLT-R (r=0.358, p <0.001), LNS (r=0.317, p<0.001), Animal naming (r=0.168, p<0.05) Social cognitive (SCOPE tests) and UPSA total: AIHQ-HB (r=-0.071, ns), AIHQ-AB (r=0.041, ns), AIHQ-BS (r=-0.005, ns), BLERT (r=0.317, p <0.001), ER-40 (r=0.360, p <0.001), Eyes (r=0.425, p <0.001), Hinting (r=0.462, p<0.001), RAD (r=0.439, p<0.001), TASIT (r=0.437, p<0.001), Trust (r=0.052, ns) Social cognitive (SCOPE tests) and SSPA average: AIHQ-HB (r=0.063, ns), AIHQ-AB (r=0.078, ns), AIHQ-BS (r=0.094, ns), BLERT (r=0.261, p <0.001), ER-40 (r=0.240, p <0.001), Eyes (r=0.300, p<0.001), Hinting (r=0.394, p<0.001), RAD (r=0.243, p<0.01), TASIT (r=0.310, p<0.01), Trust (r=-0.030, ns) | Greater NC performance in 4/5 tests (speed of processing, working memory and verbal learning) showed a significant association with greater FC performance  Greater performance on 6/8 SC tests (emotional processing, theory of mind, social perception) showed a significant association for with greater FC | 179 | Small negative relationship between UPSA total and MCCB TMT-A test; small positive relationship between UPSA total and MCCB Symbol coding test/Animal naming test; medium positive relationship between UPSA total and MCCB HVLT-R test; large positive relationship between UPSA total and LNS test  Small negative relationship between SSPA average and MCCB TMT-A test; medium positive relationship between SSPA average and MCCB Symbol coding test/HVLT-R test/LNS test; small positive relationship between SSPA average and MCCB Animal naming test  Minimal negative relationship between UPSA total and SCOPE AIHQ-HB test/AIHQ-BS test; minimal positive relationship between UPSA total and SCOPE AIHQ-AB test/Trust test; medium positive relationship between UPSA total and SCOPE BLERT test/ER-40 test/Eyes test/Hinting test/RAD test/TASIT test  Minimal positive relationship between SSPA average and SCOPE AIHQ-HB test/AIHQ-AB test/AIHQ-BS test; small positive relationship between SSPA average and SCOPE BLERT test/ER-40 test/RAD test; medium positive relationship between SSPA average and Eyes test/Hinting test/TASIT test; minimal negative relationship between SSPA average and Trust test |
| Pinkham et al.^67^ | 2018 | US | Cross-sectional | 218 | MCCB and UPSA-B MCCB and SSPA SCOPE and UPSA-B SCOPE and SSPA | MCCB subtest SCOPE subtest | Social cognitive (SCOPE tests) and UPSA-B total:  BLERT (r=0.368, p<0.001), ER-40 (r=0.361, p<0.001), Eyes (r=0.381, p<0.001), IBT (r=-0.189, p<0.01), Hinting (r=0.404, p<0.001), MiniPONS (r=0.391, p<0.001), SAT-MC (r=0.265, p<0.001), TASIT (r=0.362, p<0.001), BLERT CR (r=-0.080, ns), BLERT RT (sec) (r=−0.029, ns), ER-40 CR (r=-0.181, p<0.01), ER-40 RT (sec) (r=−0.110, ns), TASIT RT (sec) (r=−0.018, ns) Neurocognition (MCCB tests) and UPSA total : TMT-A (r=−0.291, p<0.001), Symbol Coding (r=0.388, p<0.001), HVLT-R (r=0.394, p<0.001), Letter-Number Span (r=0.423, p<0.001), Animal Naming (r=0.236, p<0.01) Social cognitive (SCOPE tests) and SSPA average:  BLERT (r=0.415, p<0.001), ER-40 (r=0.410, p<0.001), Eyes (r=0.277, p<0.001), IBT (r=−0.137, ns), Hinting (r=0.437, p<0.001), MiniPONS (r=0.379, p<0.001), SAT-MC (r=0.329, p<0.001), TASIT (r=0.380, p<0.001), BLERT CR (r=−0.030, ns), BLERT RT (sec) (r=−0.176, p<0.05), ER-40 CR (r=−0.090, ns), ER-40 RT (sec) (r=−0.292, p<0.001), TASIT RT (sec) (r=−0.105, ns) Neurocognition (MCCB tests) and SSPA average:  TMT-A (r=−0.215, p<0.01), Symbol Coding r=0.290, p<0.001), HVLT-R r=0.337, p<0.001), Letter-Number Span r=0.322, p<0.001), Animal Naming r=0.195, p<0.001)  Regression models showed that social cognitive tasks significantly accounted for 25% of the variance in UPSA-B [adjusted R^2^=0.246, F(9,188)=8.16, p<0.001] and 31% of the variance in SSPA [adjusted R^2^=0.305, F(9,191)=10.74, p<0.001] | Greater NC performance in 5/5 tests (speed of processing, working memory, and verbal learning) showed a significant association with greater FC performance   Greater performance in 8/8 SC tests (attributional style, social perception, emotion processing, theory of mind) showed a significant association with greater FC performance | 208 | Medium positive relationship between UPSA-B and BLERT, ER-40, Eyes, Hinting, MiniPONS, TASIT; small negative relationship between UPSA-B and IBT, ER-40 CR, ER-40 RT (sec); minimal negative relationship between UPSA-B and BLERT CR, BLERT RT (sec), TASIT RT (sec); small positive relationship between UPSA-B and SAT-MC social cognitive task  Small negative relationship between UPSA-B average and TMT-A neurocognitive task; medium positive relationship between UPSA-B average and Symbol Coding, HVLT-R, Letter-Number Span; small positive relationship between UPSA-B average and Animal Naming neurocognitive task  Medium positive relationship between SSPA average and BLERT, ER-40, Hinting, MiniPONS, SAT-MC, TASIT; small positive relationship between SSPA average and Eyes social cognitive task; small negative relationship between SSPA average and IBT, BLERT RT (sec), ER-40 RT (sec), TASIT RT (sec); minimal negative relationship between SSPA average and BLERT CR, ER-40 CR Small negative relationship between SSPA average and TMT-A neurocognitive task; small positive relationship between SSPA average and Symbol Coding, Animal Naming; medium positive relationship between SSPA average and HVLT-R, Letter-Number Span |
| Poletti et al.^68^ | 2012 | Italy | Cross-sectional | 62 | BACS and SCoRS | BACS subscales | ANOVA analysis of the relationship between cognitive performance (BACS) and SCoRS scores (patients/relatives/informants): Memory (F=0.089, p=0.767/F=0.608, p=0.438/F=11.556, p=0.001), attention-symbol coding (F=0.481, p=0.489/F=0.223, p=0.639/F=12.621, p<0.001), executive functions-Tower of London (F=0.384, p=0.682/F=6.824, p=0.011/F=10.750, p=0.001), psychomotor coordination (F=0.358, p=0.551/F=0.579, p=0.449/F=13.797, p<0.001) | Greater GC performance in 4/4 tests showed a significant association with informant SCoRS   Greater GC performance in 1/4 tests showed a significant association with relative SCoRS  0/4 GC tests did not show a significant association with patient SCoRS | Effect size not interpreted - ANOVA analysis | |
| Russell et al.^69^ | 2022 | US | Cross-sectional | 159 | MCCB and CAI | MCCB subtests | MCCB tests (in person/remote) and CAI overall-remote administration: TMT-A (r=0.30, p<0.01/r=0.40, p<0.05), LNS (r=-0.21, p<0.05/r=-0.49 p<0.01), Animal fluency (r=-0.23, p<0.05/r=-0.31, ns), HVLT-R (r=-0.16, ns/r=-0.37, p < 0.05) MCCB tests (in person/remote) and CAI overall-informant: TMT-A (r=0.23, p<0.05/r=0.06, ns), LNS (r=-0.08, ns/r=-0.45, p<0.05), Animal fluency (r=-0.24, p<0.05/r=-0.11, ns), HVLT-R (r=0.06, ns/r=-0.16, ns) MCCB tests (in person/remote) and CAI GAF-remote administration: TMT-A (r=-0.28, p<0.01/r= -0.37, p<0.05), LNS (r=0.27, p<0.01/r=0.41, p<0.01), Animal fluency (r=0.23, p<0.05/r=0.18, ns), HVLT-R (r=0.15, ns/r=0.30, ns) MCCB tests (in person/remote) and CAI GAF-informant: TMT-A (r=-0.22, p<0.05/r=-0.15, ns), LNS (r=0.08, ns/r=0.33, ns), Animal fluency (r=0.29, p<0.01/r=0.05, ns), HVLT-R (r=-0.16, ns/r=0.29, ns) | Greater NC processing speed showed a significant association with greater FC [ns for TMT-A remote and CAI informant, animal fluency remote, and CAI informant and CAI remote]  Greater NC verbal working memory showed a significant association with greater FC [ns for LNS in-person and CAI informant]  Greater NC verbal learning and memory showed a significant association with [ns for in-person HVLT-R for CAI-remote and in-person/remote HVLT-R] | TMT-A: n=114 (in person), n=35 (remote); LNS: n=116 (in person), n=43 (remote); Animal fluency: n=115 (in person), n=43 (remote); HVLT-R: n=114 (in person), n=43 (remote) | Medium positive relationship between CAI overall-remote administration and TMT-A MCCB test (in person and remote); small negative relationship between CAI overall-remote administration and LNS MCCB test (in person); medium negative relationship between CAI overall-remote administration and LNS MCCB test (remote); small negative relationship between CAI overall-remote administration and Animal fluency test (in person); medium negative relationship between CAI overall-remote administration and Animal fluency test (remote); small negative relationship between CAI overall-remote administration and HVLT-R test (in person); medium negative relationship between CAI overall-remote administration and HVLT-R test (remote)  Small positive relationship between CAI overall-informant and TMT-A MCCB test (in person); minimal positive relationship between CAI overall-informant and TMT-A MCCB test (remote); minimal negative relationship between CAI overall-informant and LNS MCCB test (in person); medium negative relationship between CAI overall-informant and LNS MCCB test (remote); small negative relationship between CAI overall-informant and Animal fluency MCCB test (in person); small negative relationship between CAI overall-informant and Animal fluency MCCB test (remote); minimal positive relationship between CAI overall-informant and HVLT-R MCCB test (in person); small negative relationship between CAI overall-informant and HVLT-R MCCB test (remote)  Small negative relationship between CAI GAF-remote administration and TMT-A MCCB test (in person); medium negative relationship between CAI GAF-remote administration and TMT-A MCCB test (remote); small positive relationship between CAI GAF-remote administration and LNS MCCB test (in person); medium positive relationship between CAI GAF-remote administration and LNS MCCB test (remote); small positive relationship between CAI GAF-remote administration and Animal fluency MCCB test (in person); small positive relationship between CAI GAF-remote administration and Animal fluency MCCB test (remote); small positive relationship between CAI GAF-remote administration and HVLT-R MCCB test (in person); medium positive relationship between CAI GAF-remote administration and HVLT-R MCCB test (remote)  Small negative relationship between CAI GAF-informant and TMT-A MCCB test (in person); small negative relationship between CAI GAF-informant and TMT-A MCCB test (remote); minimal positive relationship between CAI GAF-informant and LNS MCCB test (in person); medium positive relationship between CAI GAF-informant and LNS MCCB test (remote); small positive relationship between CAI GAF-informant and Animal fluency MCCB test (in person); minimal positive relationship between CAI GAF-informant and Animal fluency MCCB test (remote); small negative relationship between CAI GAF-informant and HVLT-R MCCB test (in person); small positive relationship between CAI GAF-informant and HVLT-R MCCB test (remote) |
| Silverstein et al.^70^ | 2010 | US | Cross-sectional | 155 | MCCB and UPSA |  | MCCB total battery composite score and UPSA-2 total score (r=0.42, p<0.001) | Greater GC performance showed a significant association with greater FC | 121 | Medium positive relationship between MCCB total battery composite score and UPSA-2 total score |
| Sitzer et al.^71^ | 2008 | US | Cross-sectional | 194 | DRS and SSPA |  | DRS total scores were significantly correlated with SSPA total scores (r=0.388, p<0.001) and all DRS subscales were significantly correlated with SSPA total scores | Greater NC performance showed a significant association with greater FC performance | 194 | Medium positive relationship between DRS total scores and SSPA total scores |
| Sumiyoshi et al.^72^ | 2014 | Japan | Cross-sectional | 64 | MCCB and UPSA-B |  | MCCB overall composite and the UPSA-B total score (r=0.35, p<0.01) | Greater NC performance showed a significant association with greater FC performance | 64 | Medium positive relationship between MCCB overall composite and the UPSA-B total score |
| Sumiyoshi et al.^73^ | 2016 | Japan | Cross-sectional | 58 | BACS and UPSA-B |  | BACS and UPSA-B (r=0.738, p<0.01) | GC performance showed a significant positive association with FC | 58 | Large positive relationship between BACS and UPSA-B |
| Tsoutsoulas et al.^74^ | 2016 | Canada | Cross-sectional | 60 | MCCB and UPSA |  | MCCB Global Score significantly predicted UPSA Total Score (B=0.05, SE± 0.016, β=0.36, p=0.005) | Greater GC performance significantly predicted greater FC performance | Effect size not interpreted - only regression coefficient value reported | |
| Twamley et al.^75^ | 2002 | US | Cross-sectional | 111 | DRS and UPSA |  | UPSA total scores were significantly correlated with total DRS scores (r=0.61, p<0.001) and all DRS subscales | Greater NC performance showed a significant association with greater FC performance | 111 | Large positive relationship between UPSA total scores and total DRS scores |
| Velligan et al.^76^ | 2014 | India | Cross-sectional | 141 | MCCB and UPSA-B |  | UPSA and MCCB (r=0.58, p<0.0001) | Greater GC performance showed a significant positive association with greater FC performance | 141 | Large positive relationship between UPSA and MCCB |
| Ventura et al.^80^ | 2020 | US | Cross-sectional | 42 | MCCB and UPSA-2 MCCB and VRFCAT MCCB and CAI |  | MCCB and VRFCAT total completion time  (r=-0.703, p<0.01) MCCB and VRFCAT total number of errors  (r=-0.602, p<0.01) MCCB and VRFCAT total forced progressions  (r=-0.658, p<0.01) MCCB and UPSA  (r=0.862, p<0.01) MCCB and CAI (patient/informant/rater)  (r=-0.660/-0.651/-0.718, all p<0.01) | Greater GC performance showed a significant very positive association with greater FC performance | 42 | Large negative relationship between MCCB and VRFCAT total completion time Large positive relationship between MCCB and VRFCAT total number of errors Large negative relationship between MCCB and VRFCAT total forced progressions Large positive relationship between MCCB and UPSA Large negative relationship between MCCB and CAI (patient/informant/rater) |
| Ventura et al.^79^ | 2016 | US | Cross-sectional | 98 | MCCB and UPSA MCCB and CAI |  | MCCB composite score and CAI (patient/informant/rater) (r=-0.54/-0.57/-0.60, all p<0.01) MCCB composite score and UPSA total score (r=0.77, p<0.01) Change in MCCB was significantly correlated with the change in the CAI Rater scores (r=−0.52, p=0.01) from baseline to six months | Greater GC performance showed a significant positive association with greater FC performance | MCCB and CAI, n=99; MCCB and UPSA, n=99; MCCB and change in CAI Rater scores, n=23 | Large negative relationship between MCCB composite score and CAI (patient/informant/rater) Large positive relationship between MCCB composite score and UPSA total score Large positive relationship between change in MCCB and change in the CAI Rater scores from baseline to 6 months |
| Ventura et al.^78^ | 2013 | US | Cross-sectional | 150 | MCCB and UPSA-2 MCCB and CAI |  | MCCB composite score and CAI (patient/informant/rater) (r=-0.39/-0.41/-0.40, p<0.01 for all values) MCCB composite score and UPSA total score (r=0.68, p<0.01) | Greater GC performance showed a significant positive association with greater FC performance | 146 | Medium negative relationship between MCCB composite score and CAI (patient/informant/rater) Large positive relationship between MCCB composite score and UPSA total score |
| Ventura et al.^77^ | 2010 | US | Cross-sectional | 176 | MCCB and SCoRS MCCB and CAI |  | SCoRS and MCCB (patient/informant/rater)  (r=-0.18/-0.21/-0.31, all ns) CAI and MCCB (patient/informant/rater)  (r=-0.28/-0.30/-0.32, all p<0.01) | Greater GC performance showed a significant association with greater FC performance [ns for SCoRS] | 172 | Small negative relationship between MCCB and SCoRS (patient/informant) Medium negative relationship between MCCB and SCoRS (rater composite) Small negative relationship between MCCB and CAI (patient) Medium negative relationship between MCCB and CAI (informant/rater composite) |
| Vesterager et al.^81^ | 2012 | Denmark | Longitudinal | 117 | MCCB and UPSA-B |  | The MCCB composite score (B=0.50, p<0.001) and all MCCB domains were significantly associated with functional capacity at baseline  Working memory was the strongest predictor of functional capacity (B=0.50, p<0.001) | Greater GC performance showed a significant association with greater FC performance | Effect size not interpreted - only regression coefficient value reported | |
| Wang et al.^82^ | 2016 | Taiwan | Cross-sectional | 66 | BACS and UPSA-B |  | BACS composite score and UPSA-B scores (r=0.70, p<0.001) | Greater GC performance showed a significant association with greater FC performance | 66 | Large positive relationship between BACS composite score and UPSA-B scores |
| Yang et al.^83^ | 2021 | Singapore | Cross-sectional | 58 | BACS and UPSA-B |  | There was a moderate correlation between BACS and UPSA-B (r=0.459, p<0.001) | Greater GC performance showed a significant association with greater FC performance | 58 | Medium positive relationship between BACS and UPSA-B |
| **Interventional studies** | | | | | | | | | | |
| Flores et al.^86^ | 2022 | US | RCT | 86 | MCCB and UPSA-B |  | Improvements in overall cognition (MCCB) significantly mediated CET-related gains in functional capacity at mid-treatment at 9 months (B=2.42, p=0.006) and treatment completion at 18 months (B=1.96, p=0.042) | Greater GC performance significantly mediated CET-related gains in FC | Effect size not interpreted - only regression coefficient value reported | |
| Harvey et al.^87^ | 2013 | 24 centers in the US (n=151), 10 in Russia (n=87),10 in India (n=98), 9 in Ukraine (n=75), 6 in Romania (n=49), 4 in Colombia (n=26) | RCT | 488 | CogState and UPSA-B |  | Significant cross-sectional correlation between baseline cognitive composite Z-score (CogState) and UPSA-B in both full and evaluable analysis samples (p<0.001) Significant longitudinal association between change in UPSA-B and change in cognitive performance: full analysis sample (p<0.001), evaluable analysis sample (P=0.022); relationship increased over time (p<0.042) and was similar across the treatment groups (all p values>0.206) | Greater GC showed a significant association with greater FC | Effect size not interpreted - no r value | |
| Javitt et al.^88^ | 2012 | US | RCT | 69 | MCCB and UPSA |  | Baseline MCCB composite and UPSA scores were strongly correlated (r=0.77, p<0.05) | Greater NC showed a significant strong association with greater FC | 57 | Large positive relationship between baseline MCCB composite and UPSA scores |
| Marx et al.^89^ | 2014 | Singapore | RCT | 120 | MCCB and UPSA-B |  | UPSA-B and the MCCB composite scores: 6-domain MCCB: Week 2 (randomization visit) r_s_=0.55, p<0.0001 (n=110); Week 6: r_s_=0.55, p<0.0001 (n=110); Week 10: r_s_=0.46, p<0.0001 (n=108) 7-domain MCCB: Week 2 (randomization visit): 6 r_s_=0.57, p<0.0001 (n=109); Week 6: r_s_=0.60, p<0.0001 (n=110); Week 10: r_s_=0.51, p<0.0001 (n=108)  The correlation between the UPSB-B and MCCB did weaken somewhat in the current investigation at completion compared to baseline (r=0.46 at completion vs r=0.55 at study entry) | Greater NC showed a significant moderate-strong association with greater FC | Week 2: n=110 for 6-domains MCCB; n=109 for 7-domains MCCB Week 6: n=110 Week 10: n=108 | Large positive relationship between UPSA-B and the MCCB composite scores (6-domain MCCB) at Week 2 and at Week 6 Medium positive relationship between UPSA-B and the MCCB composite scores (6-domain MCCB) at Week 10 Large positive relationship between UPSA-B and the MCCB composite scores (7-domain MCCB) at Week 2, Week 6, and Week 10 |
| Medalia et al.^90^ | 2019 | US | RCT | 103 | MCCB and UPSA-B |  | Change in MCCB was significantly correlated with change in UPSA-B measured from baseline to follow-up (r=0.55, p<0.001) | Greater NC showed a significant moderate association with greater FC | 61 | Large positive relationship between change in MCCB and change in UPSA-B measured from baseline to follow-up |
| Miley et al.^91^ | 2020 | US | RCT | 111 | MCCB and UPSA-B |  | Changes in the UPSA-Brief from baseline to 6-month follow-up were significantly associated with changes in Global Cognition from baseline to completion of the auditory training module at 40 h of training (r=0.44, p<0.01), and from baseline to post training (r=0.35, p=0.05) | Greater NC showed a significant weak-moderate association with greater FC | 34 | Medium positive relationship between changes in the UPSA-Brief from baseline to 6-month follow-up and changes in Global Cognition from baseline to completion of the auditory training module at 40 h of training Medium positive relationship between changes in the UPSA-Brief from baseline to 6-month follow-up and changes in Global Cognition from baseline to post training |
| Saperstein et al.^92^ | 2021 | US | Other interventional | 132 | MCCB and UPSA |  | Improvement in UPSA significantly associated with better baseline MCCB (p<0.001) | Greater NC showed a significant association with greater FC | Effect size not interpreted - no r value | |
| Tan et al.^93^ | 2020 | China | RCT | 311 | MCCB and UPSA |  | Improvement in MCCB total score was marginally associated with improvement in UPSA (F=3.01, p=0.09) | Greater GC showed a non-significant association with greater FC | Effect size not interpreted - only F value reported | |
| Zhu et al.^94^ | 2020 | China | RCT | 157 | MCCB and UPSA |  | Improvement in MCCB total score was significantly associated with UPSA score (F=12.94, p=0.00) | Greater NC showed a significant association with greater FC | Effect size not interpreted - only F value reported | |

^a^Number of individuals with a diagnosis of schizophrenia, schizophrenia spectrum disorders and first episode non-affective psychosis.

^b^Analysis of unpublished data obtained directly from the study authors.

AIHQ, Ambiguous Intentions Hostility Questionnaire; AIHQ-AB, Ambiguous Intentions Hostility Questionnaire – Aggression bias; AIHQ-BS, Ambiguous Intentions Hostility Questionnaire – Blame score; AIHQ-HB, Ambiguous Intentions Hostility Questionnaire – Hostility bias; BACS, Brief Assessment of Cognition in Schizophrenia; BLERT, Bell Lysaker Emotion Recognition Task; CAI, Cognitive Assessment Interview; CogState, CogState Schizophrenia Battery; DRS, Dementia Rating Scale; ER-40, Penn Emotion Recognition Task; FC, functional capacity; GC, global cognition; MASC, Maryland Assessment of Social Competence; MCCB, MATRICS Consensus Cognitive Battery; MMAA, Multimodal Attention Assessment Battery; NC, neurocognition; ns, non-significant; QLS, Quality of Life Scale; RCT, randomized controlled trial; RAD, Relationships Across Domains; RBANS, Repeatable Battery for the Assessment of Neuropsychological Status; SCOPE, Social Cognition Psychometric Evaluation; SCoRS, Schizophrenia Cognition Rating Scale; SSPA, Social Skills Performance Assessment; TASIT, The Awareness of Social Inferences Test; UPSA, UCSD Performance-Based Skills Assessment; UPSA-B, UCSD Performance-Based Skills Assessment - Brief version; US, United States.

### *Supplementary Table 6. Strength of the relationship between cognitive performance and functional capacity across instrument pairs (n=63)*

| **Instrument pair** | | **No. studies** | **Relationship between overall instrument scores** | | | | | **Relationship between subtest/domain instrument scores*** | | | | |
| --- | --- | --- | --- | --- | --- | --- | --- | --- | --- | --- | --- | --- |
|  |  |  | **No. studies with relevant effect size data** | **Strength** | | | | **No. studies with relevant effect size data** | **Strength** | | | |
| **Cognitive performance** | **Functional capacity** |  |  | **Minimal**  r<0.10 d<0.2 | **Small**  0.1≤r<0.3 0.20≤d<0.5 | **Medium**  0.3≤r<0.5 0.5≤d<0.8 | **Large**  r≥0.5 d≥0.8 |  | **Minimal**  r<0.10 d<0.2 | **Small**  0.1≤r<0.3 0.20≤d<0.5 | **Medium**  0.3≤r<0.5  0.5≤d<0.8 | **Large**  r≥0.5  d≥0.8 |
| MCCB | UPSA/UPSA-B | 40^30,33,35,36,39–44,48,51–53,55–57,61–64,66,67,70,72,74,76,78–81,84,86,88–94^ | 27^30,33,39,40,42,43,48,52,53,55–57,62–64,66,70,72,76,78–80,84,88–91^ | 0 | 0 | 6^33,56,70,72,84,91^ | 22^30,39,40,42,43,^  ^48,52,53,^  ^55,57,62–66,76,78–80,88–90^ | 2^36,67^ | 1^67^ | 2^36,67^ | 2^36,67^ | 1^36^ |
| MCCB | CAI | 8^30,42,53,69,^  ^77–80^ | 7^30,42,53,77–80^ | 0 | 2^30,53^ | 1^77^ | 4^42,78–80^ | 1^69^ | 0 | 1^69^ | 1^69^ | 0 |
| MCCB | SSPA | 8^33,36,44,56,57,63,67,84^ | 5^33,56,57,63,84^ | 0 | 2^84^ | 4^33,56,57,63^ | 0 | 2^36,67^ | 0 | 2^36,67^ | 2^36,67^ | 0 |
| BACS | UPSA/UPSA-B | 6^37,47,54,^  ^73,82,83^ | 5^37,54,73,82,83^ | 0 | 0 | 2^37,83^ | 3^54,73,82^ | 1^47^ | 0 | 1^47^ | 1^47^ | 0 |
| BACS | SCoRS | 6^38,45,54,59,^  ^68,86^ | 5^38,45,54,59,86^ | 0 | 1^38^ | 1^45^ | 3^54,59,85^ | 0 | 0 | 0 | 0 | 0 |
| MCCB | SCoRS | 4^43,49,77,95^ | 4^43,49,77,95^ | 0 | 1^77^ | 1^95^ | 2^43,49^ | 0 | 0 | 0 | 0 | 0 |
| SCOPE | UPSA/UPSA-B | 3^36,56,67^ | 0 | 0 | 0 | 0 | 0 | 2^36,67^ | 2^36,67^ | 1^67^ | 2^36,67^ | 0 |
| MCCB | VRFCAT | 2^52,80^ | 2^52,80^ | 0 | 0 | 0 | 2^52,80^ | 0 | 0 | 0 | 0 | 0 |
| MCCB | MASC | 2^43,64^ | 2^43,64^ | 0 | 1^43^ | 2^43,64^ | 0 | 0 | 0 | 0 | 0 | 0 |
| DRS | SSPA | 2^71,148^ | 2^71,148^ | 0 | 0 | 1^71^ | 1^148^ | 0 | 0 | 0 | 0 | 0 |
| CogState | UPSA/UPSA-B | 2^66,87^ | 1^66^ | 0 | 0 | 0 | 1^66^ | 0 | 0 | 0 | 0 | 0 |
| DRS | UPSA | 2^58,75^ | 1^75^ | 0 | 0 | 0 | 1^75^ | 0 | 0 | 0 | 0 | 0 |
| SCOPE | SSPA | 1^67^ | 1^67^ | 1^67^ | 1^67^ | 1^67^ | 0 | 0 | 0 | 0 | 0 | 0 |

*Individual studies can contribute to more than 1 category if multiple domains are assessed in a single instrument.

Instrument pairs with no effect size data for comparisons: RBANS and UPSA/UPSA-B, DRS and MMAA, SCOPE, and SLOF.

BACS, Brief Assessment of Cognition in Schizophrenia; CAI, Cognitive Assessment Interview; CogState, CogState Schizophrenia Battery; DRS, Dementia Rating Scale; MASC, Maryland Assessment of Social Competence; MCCB, MATRICS Consensus Cognitive Battery; MMAA, Multimodal Attention Assessment Battery; RBANS, Repeatable Battery for the Assessment of Neuropsychological Status; SCOPE, Social Cognition Psychometric Evaluation; SCoRS, Schizophrenia Cognition Rating Scale; SLOF, Specific Level of Functioning scale; SSPA, Social Skills Performance Assessment; UPSA, UCSD Performance-Based Skills Assessment; UPSA-B, UCSD Performance-Based Skills Assessment - Brief version; VRFCAT, Virtual Reality Functional Capacity Assessment Tool.

### *Supplementary Table 7. Characteristics and key results of final included studies examining the relationship between cognitive performance and real-world functioning (n=43)*

| **Author** | **Publication Year** | **Country (stated or clear from authorship)** | **Study design** | **Sample size^a^** | **Instrument pair(s)** | **Subtest/domain score (overall/total score not reported)** | **Key results** | **Interpretation of association** | | |
| --- | --- | --- | --- | --- | --- | --- | --- | --- | --- | --- |
|  |  |  |  |  |  |  |  | **Significance of association** | **Effect size** | |
|  |  |  |  |  |  |  |  |  | **N, analysis** | **Strength of association** |
| **Observational studies** | | | | | | | | | | |
| Bechi et al.^37^ | 2017 | Italy | Cross-sectional | 79 | BACS and QLS |  | BACS total and QLS total (r=0.234, p=0.039) | Greater NC performance showed a significant association with greater RWF performance | 79 | Small positive relationship between BACS and QLS |
| Bergh et al.^96^ | 2016 | Denmark | Longitudinal | 273 | BACS and real-world milestones | Milestone (employment) | BACS at 10 years predicted by baseline unemployment in univariate analysis (β=-0.43, CI:  -0.65 to -0.21, p<0.001) | Unemployment was significantly associated with worse GC performance | Effect size not interpreted - only regression coefficient value reported | |
| Engelstad et al.^97^ | 2017 | Norway | Cross-sectional | 54 | MCCB and SFS |  | MCCB and SFS (r=0.00, ns) | FC performance showed no association with RWF performance | 54 | No relationship between MCCB and SFS |
| Fervaha et al.^39^ | 2015 | US | Cross-sectional | 176 | MCCB and real-world milestones | Milestone (employment) | MCCB and employment status (r=0.29, p<0.001) | Greater NC performance showed a significant association with greater RWF performance | 176 | Small positive relationship between MCCB and employment status |
| Fujino et al.^40^ | 2016 | Japan | Cross-sectional | 93 | MCCB and SFS |  | MCCB significantly predicted SFS total score (r=0.423, p<0.01) MCCB significantly predicted SFS employment domain (r=0.388, p<0.01) | Greater GC performance showed a significant association with greater RWF performance | 93 | Medium positive relationship between MCCB and SFS total score; medium positive relationship between MCCB and SFS employment domain |
| Galderisi et al.^41^ | 2016 | Italy | Cross-sectional | 921 | MCCB and SLOF | SLOF subscales | NC MCCB showed significant indirect effects on SLOF interpersonal relationships through social cognition or FC (β=0.116, p<0.001) NC MCCB showed significant indirect effects on SLOF work skills through social cognition or FC (β=0.212, p<0.001) | Greater NC did not show a significant direct association with RWF performance in interpersonal relationships or work skills, only an indirect relationship through SC | Effect size not interpreted - only regression coefficient value reported | |
| Giordano et al.^42^ | 2022 | Italy | Cross-sectional | 580 | MCCB and SLOF | SLOF subscales | MCCB composite score and SLOF scales: interpersonal relationship (r=0.244, p<0.0004), everyday life skills (r=0.451, p<0.0004), working skills (r=0.440, p<0.0004) | Greater GC performance showed a significant association with greater RWF interpersonal relationship and a significant association with greater RWF everyday life skills and RWF working skills | 580 | Small positive relationship between MCCB composite score and SLOF interpersonal relationship scale; medium positive relationship between MCCB composite score and SLOF everyday life skills scale; medium positive relationship between MCCB composite score and SLOF working skills scale |
| Gold et al.^98^ | 1999 | US | Cross-sectional | 129 | RBANS and real-world milestones | Milestone(employment) | Significant difference (p<0.01, t-tests) between employed and unemployed patient groups in total RBANS score, and 4/5 index scores. Remained significant after controlling for years of education. | GC was significantly associated with employment outcome | Effect size not interpreted - only t-test outcomes reported | |
| Gould et al.^99^ | 2015 | US | Cross-sectional | 214 | MCCB and SLOF | SLOF subscales | Predictive value of MCCB overall performance (global NP T score) for SLOF subscales in step-wise regression: Activities subscale, step 2 R^2^=0.26, p<0.001 | Greater NC performance was significantly associated with greater RWF activities performance but not other SLOF domains | Effect size not interpreted - only R squared value reported | |
| Hajdúk et al.^44^ | 2021 | US | Cross-sectional | 408 | MCCB and SLOF |  | MCCB did not show a strong direct relationship with SLOF but did show an indirect relationship through functional competence (UPSA) and social competence (SSPA) | Greater NC performance did not show a direct relationship with RWF performance but showed an indirect relationship through FC | Effect size not interpreted - network analysis | |
| Ho et al.^46^ | 2013 | US | Cross-sectional | 138 | RBANS and SLOF | SLOF subscales | RBANS did not significantly predict SLOF activities after controlling for the UPSA  (B=0.071 ±0.071, ns)  RBANS did not significantly predict SLOF work skills after controlling for the UPSA  (B=-0.004 ±0.044, ns) | GC performance did not significantly predict RWF performance in everyday activities or work skills after controlling for FC performance | Effect size not interpreted - only regression coefficient value reported | |
| Kim et al.^100^ | 2021 | Korea | Cross-sectional | 49 | MCCB and WHODAS |  | MCCB composite score and WHODAS overall score  (r=-0.194, ns) | Greater GC showed a non-significant association with greater RWF performance | 49 | Small negative relationship between MCCB composite score and WHODAS overall score |
| Kurtz et al.^101^ | 2018 | India | Longitudinal | 60 | MCCB and WHODAS |  | MCCB (mean cognition, at study entry) and WHODAS overall percentile (1 year follow-up)  (r=-0.20, ns) | Greater NC showed a non-significant association with greater RWF performance | 60 | Small negative relationship between MCCB (mean cognition, at study entry) and WHODAS overall percentile (1 year follow-up) |
| Lin et al.^102^ | 2013 | China | Cross-sectional | 302 | MCCB and QLS | MCCB subtests | All 7 MCCB domains (speed of processing, attention, working memory, verbal learning and memory, visual learning and memory, reasoning and problem solving, social cognition) showed significant correlation with QLS (p<0.01)  The basic model depicted the direct relationship between MCCB and QLS as statistically significant (standardized coefficient β=0.67, p<0.001) The significant direct relationship between neuro-/social cognition and functional outcome was no longer significant, as soon as the mediator of clinical symptoms was entered into the model (β=0.21, p=0.076) | Greater GC performance showed a significant association with greater RWF performance | Effect size not interpreted - only regression coefficient value reported | |
| Llerena et al.^103^ | 2018 | US | Cross-sectional | 76 | MCCB and real-world milestones | Milestone (living status) | Days unsheltered were correlated with overall cognition such that poorer overall cognition was associated with more days unsheltered (r=-0.388, p=0.008). | Greater GC performance showed a significant association with fewer days unsheltered | 30 | Medium negative relationship between overall cognition and days unsheltered |
| Ludwig et al.^56^ | 2017 | US | Cross-sectional | 38 | MCCB and SLOF SCOPE and SLOF | SLOF subscales | MCCB composite and SLOF self-report average (r=0.123, ns) SCOPE social cognitive tasks and SLOF self-report average: significant correlation (p<0.05) for AIHQ-HB, AIHQ-BS, Eyes [ns for SLOF informant] In regression model, MCCB composite was not a significant predictor of SLOF self-report values | Greater NC performance showed a non-significant association with greater RWF performance  Greater performance on 2/8 SC tests (attributional style, theory of mind) showed a significant association with greater RWF performance. 6/8 showed ns association | 38 | Small positive relationship between MCCB composite and SLOF self-report average |
| Mahmood et al.^57^ | 2022 | US | Cross-sectional | 55 | MCCB and SLOF |  | MCCB and SLOF (r=0.22, p=0.114) | Greater GC showed a non-significant association with greater RWF performance | 55 | Small positive relationship between MCCB and SLOF |
| Mausbach et al.^58^ | 2008 | US | Cross-sectional | 434 | DRS and real-world milestones | Milestone (living status) | DRS was a poor predictor of independent living status score (ROC AUC=0.65; 95% CI: 0.58–0.73) | NC performance was a poor predictor of independent living status | Effect size not interpreted - only ROC AUC value reported | |
| Miller et al.^33^ | 2021 | US | Cross-sectional | 557 | MCCB and real-world milestones | Milestones (living status and financial independence) | MCCB subtest and residential independence (Y/N): t=3.69, p<0.001, Cohen's d=0.45^b^  MCCB subtest and financial independence (Y/N): t=4.24, p<0.001, Cohen's d=0.39^b^ | Greater GC showed a significant association with greater RWF performance | 480 | Small difference in MCCB subtest by residential independence status  Small difference in MCCB subtest by financial independence status |
| Mucci et al.^61^ | 2021 | Italy | Longitudinal | 618 | MCCB and SLOF | SLOF subscales | Higher baseline neurocognitive functioning (MCCB) was associated, as measured in the model by standardized regression coefficients, with positive change at 4-year follow-up in SLOF everyday life skills (β=0.370; 95% CI 0.253 to 0.486; p<0.001) and SLOF work skills (β=0.102; 95% CI 0.016 to 0.188; p=0.02) | GC performance showed a significant positive association with RWF performance in everyday life skills and work skills but not interpersonal relationships | Effect size not interpreted - only regression coefficient value reported | |
| Nagendra et al.^63^ | 2020 | US | Cross-sectional | 169 | MCCB and SLOF |  | MCCB composite and SLOF informant rating (r=0.28, p<0.01) | NC performance showed a significant positive association with RWF informant rated performance | 169 | Small positive relationship between MCCB composite and SLOF informant rating |
| Oliver et al.^104^ | 2019 | US | Cross-sectional | 164 | MCCB and QLS |  | Significant direct effect of neurocognition (MCCB) on QLS | NC had a significant direct effect on RWF | Effect size not interpreted - SEM model | |
| Oomen et al.^105^ | 2023 | The Netherlands | Longitudinal | 204 | BACS and WHODAS |  | No significant differences in WHODAS were present between the three cognitive cluster groups (preserved cognition, moderately impaired, severely impaired) (p>0.05) | No significant association between NC and RWF | Effect size not interpreted - no r value | |
| Pinkham et al.^36^ | 2016 | US | Cross-sectional | 179 | MCCB and SLOF SCOPE and SLOF | MCCB subtest SCOPE subtest | Neurocognition (MCCB tests) and SLOF total: TMT-A (r=-0.237, p<0.01), Symbol coding (r=0.263, p<0.001), HVLT-R (r=0.174, p<0.05), LNS (r=0.255, p<0.01), Animal naming (r=0.078, ns) Social cognitive (SCOPE tests) and SLOF total: AIHQ-HB (r=-0.058, ns), AIHQ-AB (r=-0.071, ns), AIHQ-BS (r=-0.137, ns), BLERT (r=0.310, p <0.001), ER-40 (0.046, ns), Eyes (r=0.127, ns), Hinting (r=0.197, p<0.01), RAD (r=0.202, p<0.01), TASIT (r=0.304, p<0.001), Trust (r=0.043, ns) | Greater NC performance in 4/5 tests (speed of processing, working memory and verbal learning) showed a significant association with greater RWF performance [ns for animal naming (speed of processing) and SLOF total]  Greater performance in 4/8 SC (emotional processing, theory of mind, social perception) tests showed a significant - association with greater RWF performance | 179 | Small negative relationship between SLOF total and MCCB TMT-A test Small positive relationship between SLOF total and MCCB Symbol coding test/MCCB HVLT-R test/MCCB LNS test Minimal positive relationship between SLOF total and MCCB Animal naming test Minimal negative relationship between SLOF total and SCOPE AIHQ-HB test/SCOPE AIHQ-AB test Small negative relationship between SLOF total and SCOPE AIHQ-BS test Medium positive relationship between SLOF total and SCOPE BLERT test/SCOPE TASIT test Minimal positive relationship between SLOF total and SCOPE ER-40 test/SCOPE Trust test Small positive relationship between SLOF total and SCOPE Eyes test/SCOPE Hinting test/SCOPE RAD test |
| Pinkham et al.^67^ | 2018 | US | Cross-sectional | 218 | MCCB and SLOF SCOPE and SLOF | MCCB subtest SCOPE subtest | Social cognitive (SCOPE tests) and SLOF Community Informant/SLOF-HQ Community Informant: BLERT (r=0.208, p<0.05/r=0.062, ns), ER-40 (r=0.174, p<0.05/r=0.088, ns), Eyes (r=0.154, ns/r=0.086, ns), IBT (r=−0.191, p<0.05/r=−0.004, ns), Hinting (r=0.192, p<0.05/r=0.345, p<0.05), MiniPONS (r=0.169, p<0.05/r=0.092, ns), SAT-MC (r=−0.004, ns/r=−0.028, ns), TASIT (r=0.106, ns/r=−0.016, ns), BLERT CR (r=0.060, ns/r=−0.412, p<0.01), BLERT RT (sec) (r=−0.102, ns/r=0.062, ns), ER-40 CR (r=−0.030, ns/r=−0.371, p<0.01), ER-40 RT (sec) (r=0.043, ns; r=-0.167, ns), TASIT RT (sec) (r=0.089, ns/r=−0.046, ns) Neurocognition (MCCB tests) and SLOF Community Informant/SLOF-HQ Community Informant: TMT-A (r=0.022, ns/r=−0.100, ns), Symbol Coding (r=0.095, ns/r=0.255, ns), Hopkins Verbal Learning Test-Revised (r=0.198, p<0.05/r=0.328, p<0.05), Letter-Number Span (r=0.217, p<0.05/r=0.096, p<0.050), Animal Naming (r=0.042, ns/r=0.026, ns) | Greater NC performance in 2/5 tests (working memory and verbal learning) showed a significant - association with greater RWF performance  Greater performance in 5/8 SC tests (emotion processing, attributional style, social perception, theory of mind) showed a significant - association with greater FC performance | SLOF Community Informant, n=135; SLOF-HQ Community Informant, n=53 | Minimal positive relationship between SLOF Community Informant and TMT-A neurocognitive task; small negative relationship between SLOF-HQ Community Informant and TMT-A neurocognitive task Minimal positive relationship between SLOF Community Informant and Symbol Coding neurocognitive task; small positive relationship between SLOF-HQ Community Informant and Symbol Coding neurocognitive task Small positive relationship between SLOF Community Informant and HVLT-R neurocognitive task; medium positive relationship between SLOF-HQ Community Informant and HVLT-R neurocognitive task Small positive relationship between SLOF Community Informant and Letter-Number Span neurocognitive task; positive relationship between SLOF-HQ Community Informant and Letter-Number Span neurocognitive task Minimal positive relationship between SLOF Community Informant/SLOF-HQ Community Informant and Animal Naming neurocognitive task |
| Russell et al.^69^ | 2022 | US | Cross-sectional | 159 | MCCB and SLOF | MCCB subtests | MCCB tests (in person/remote) and SLOF-remote administration: TMT-A (r=-0.19, p<0.05/r=-0.04, ns), LNS (r=0.28, p<0.01/r=0.23, ns), Animal fluency (r=0.09, ns/r=0.09, ns), HVLT-R (r=0.16, ns/r=0.26, ns) MCCB tests (in person/remote) and SLOF-informant: TMT-A (r=-0.15, ns/r=-0.10, ns), LNS (r=0.25, p<0.05/r=0.05, ns), Animal fluency (r=0.25, p<0.05/r=0.001, ns), HVLT-R (r=0.001, ns/r=0.07, ns) | Greater processing speed showed a significant association with RWF [ns for remote TMT-A and in-person/remote animal fluency for SLOF-remote and ns for in-person/remote TMT-A and remote animal fluency and SLOF-informant and tests]  Greater working memory showed a significant association with RWF [ns for remote LNS for SLOF-remote and SLOF-informant]  Greater verbal learning and memory showed a non-significant association with RWF | TMT-A: n=114 in person, n=35 remote; LNS: n=116 in person, n=43 remote; Animal fluency: n=115 in person, n=43 remote; HVLT-R: n=114 in person, n=43 remote | Small negative relationship between SLOF-remote administration and TMT-A MCCB test (in person); minimal negative relationship between SLOF-remote administration and TMT-A MCCB test (remote) Small positive relationship between SLOF-remote administration and LNS MCCB test (in person); small positive relationship between SLOF-remote administration and LNS MCCB test (remote) Minimal positive relationship between SLOF-remote administration and Animal fluency MCCB test (in person); minimal positive relationship between SLOF-remote administration and Animal fluency MCCB test (remote) Small positive relationship between SLOF-remote administration and HVLT-R MCCB test (in person); small positive relationship between SLOF-remote administration and HVLT-R MCCB test (remote) Small negative relationship between SLOF-informant and TMT-A MCCB test (in person); small negative relationship between SLOF-informant and TMT-A MCCB test (remote) Small positive relationship between SLOF-informant and LNS test (in person); minimal positive relationship between SLOF-informant and LNS test (remote) Small positive relationship between SLOF-informant and Animal fluency test (in person); minimal positive relationship between SLOF-informant and Animal fluency test (remote) Minimal positive relationship between SLOF-informant and HVLT-R test (in person); minimal positive relationship between SLOF-informant and HVLT-R test (remote) |
| Shamsi et al.^106^ | 2011 | US | Cross-sectional | 185 | MCCB and SAS | MCCB subtests SAS subscales | Working memory was a significant predictor of work/education functioning (B=0.035 ±0.017, p=0.044) Verbal learning memory is a significant predictor of independent living (B=0.050 ±0.025, p=0.048) Social functioning was predicted by social cognition (B=0.039 ±0.017, p=0.027) and attention (B=-0.035 ±0.018, p=0.054) | Greater working memory showed a significant association of greater RWF work/education functioning Greater verbal learning memory showed a significant association with independent living Greater SC and attention showed a significant association with social functioning | Effect size not interpreted - only regression coefficient value reported | |
| Silverstein et al.^70^ | 2010 | US | Cross-sectional | 155 | MCCB and SFS |  | MCCB total battery composite score and SFS total score (r=0.05, p=0.61) | Greater GC showed a non-significant association with greater RWF performance | 131 | Minimal positive relationship between MCCB total battery composite score and SFS total score |
| Starzer et al.^117^ | 2024 | Denmark | Longitudinal | 156 | BACS and real-world milestones | Milestones (employment, relationship status, living status) | Bivariate logistical regression analysis, 20-year outcomes: participants with higher neurocognitive levels (BACS) were significantly more likely to be in a relationship (OR: 1.56, CI 1.1–2.2, p=0.013), be employed (OR:2.07, CI 1.35–3.17, p<0.001), be "independent living" (OR: 2.06, CI 0.72–5.91, p=0.178) | Participants with greater GC performance were significantly more likely to be in a relationship or be employed | Effect size not interpreted - only OR value reported | |
| Strassnig et al.^107^ | 2018 | US | Cross-sectional | 821 | MCCB and SLOF | SLOF subscales | Cognitive test performance in overall sample was correlated with interpersonal relationships, everyday activities, and work skills: shared variance for these correlations was 1%, 12%, and 26% respectively across the 3 functional domains (also reported for impaired vs not impaired) | GC performance predicted RWF interpersonal relationships, everyday activities, and work skills: shared variance for these correlations was 1%, 12%, and 26% respectively | Effect size not interpreted - no r value | |
| Sumiyoshi et al.^73^ | 2016 | Japan | Cross-sectional | 58 | BACS and SLOF BACS and SFS |  | BACS and SLOF (r=0.564, p<0.01) BACS and SFS (r=0.282, p<0.1) | Greater GC performance showed an association with greater RWF performance [ns for SFS] | 58 | Large positive relationship between BACS and SLOF Small positive relationship between BACS and SFS |
| Takahashi et al.^108^ | 2017 | Japan | Cross-sectional | 45 | BACS and QLS |  | QLS score and BACS mean score (r=0.06, p=0.685) | Greater GC performance showed a non-significant association with greater RWF performance | 45 | Minimal positive relationship between QLS score and BACS mean score |
| Takeda et al.^109^ | 2019 | Japan | Cross-sectional | 36 | BACS and QLS |  | BACS composite score and QLS total (r=0.45 p=0.02)  Generalized linear model analysis showed that the common objects and activities score on the QLS was predicted by the composite BACS score (β=0.15, p=0.02) | Greater GC performance showed a significant association with greater RWF performance | 36 | Medium positive relationship between BACS composite score and QLS total |
| Tominaga et al.^110^ | 2018 | Japan | Cross-sectional | 55 | MCCB and QLS |  | MCCB composite score did not significantly correlate with QLS total (r=0.24, p>0.05) or domain scores | Greater GC performance showed a non-significant association with greater RWF performance | 55 | Small positive relationship between MCCB composite score and QLS total |
| Twamley et al.^75^ | 2002 | US | Cross-sectional | 111 | DRS and real-world milestones | Milestone (living status) | DRS total score was significantly correlated and independence (r_s_=0.30, p<0.001) | Greater NC performance showed a significant association with greater RWF performance | 111 | Medium positive relationship between DRS total score and independence |
| Ueoka et al.^111^ | 2011 | Japan | Cross-sectional | 61 | BACS and QLS |  | The BACS composite score (r=0.341, p<0.05), showed significant positive correlations with the QLS total and the QLS interpersonal relations (r=0.346, p<0.05) and intrapsychic foundation (r=0.341, p<0.05) subscales | Greater GC performance showed a significant association with greater RWF performance | 61 | Medium positive relationship between BACS composite score and QLS total Medium positive relationship between BACS composite score and QLS interpersonal relations subscale Medium positive relationship between BACS composite score and QLS intrapsychic foundation subscale |
| Velligan et al.^76^ | 2014 | India | Cross-sectional | 141 | MCCB and SLOF |  | SLOF and MCCB (r=0.50, p<0.0001) | Greater GC performance showed a significant positive association with greater RWF performance | 141 | Large positive relationship between SLOF and MCCB |
| Vesterager et al.^81^ | 2012 | Denmark | Longitudinal | 117 | MCCB and real-world milestones | Milestones (living status and financial independence) | Composite MCCB was not a predictor of real-world measures of functioning in univariate analysis: financial independence (B=0.01, p=0.56), independent living (B=0.01, p=0.78), global functioning (B=0.02, p=0.27) | GC was not a significant predictor of RWF performance in financial independence, independent living or global functioning | Effect size not interpreted - only regression coefficient value reported | |
| **Interventional studies** | | | | | | | | | | |
| Bosia et al.^112^ | 2017 | Italy | Other interventional | 100 | BACS and QLS |  | Cognitive index did not have a significant effect on QLS (OR: 0.93, p=0.99) | NC did not show a significant association with RWF | Effect size not interpreted - only regression coefficient value reported | |
| Fisher et al.^113^ | 2010 | US | RCT | 32 | MCCB and QLS | MCCB subtests | The Pearson correlations between change in cognition and change in QLS total revealed significant positive associations • Change in speed of processing and QLS total (mean item score): R^2^=0.30 (n=22) • Change in cognitive control and QLS total: R^2^=0.47 (n=22) | Greater GC performance showed a moderate-strong association with greater RWF | Effect size not interpreted - only OR reported | |
| John et al.^114^ | 2017 | Australia | Other interventional | 89 | BACS and real-world milestones | Milestone (employment) | The effect of cognitive improvement on positive employment outcome was small in magnitude, but was statistically significant (OR=1.08, p=0.015), | Greater NC showed a small but significant association with employment outcome | Effect size not interpreted - no r value | |
| Lystad et al.^115^ | 2017 | Norway | RCT | 131 | MCCB and real-world milestones | Milestone (employment) | Positive neurocognitive change did not predict occupational status at follow-up in either group | Greater NC did not show an association with occupational status | Effect size not interpreted - no r value | |
| Shimada et al.^116^ | 2022 | Japan | RCT | 136 | BACS and SFS |  | Overall BACS composite score was not significantly associated with SFS total score. SFS total score significantly associated with BACS motor speed (B=3.27, SE=1.28, *B*=0.22, t=2.56, p=0.012, 95% CI: 0.73–5.82) and BACS executive function (B=3.92, SE=1.62, *B*=0.22, t=2.42, p=0.017, 95% CI: 0.71–7.12) score but not verbal memory, working memory, verbal fluency, or attention | Overall GC did not show a significant association with RWF | Effect size not interpreted - no r value | |

^a^Number of individuals with a diagnosis of schizophrenia, schizophrenia spectrum disorders and first episode non-affective psychosis.

^b^Analysis of unpublished data obtained directly from the study authors.

AIHQ, Ambiguous Intentions Hostility Questionnaire; AIHQ-AB, Ambiguous Intentions Hostility Questionnaire – Aggression bias; AIHQ-BS, Ambiguous Intentions Hostility Questionnaire – Blame score; AIHQ-HB, Ambiguous Intentions Hostility Questionnaire – Hostility bias; BACS, Brief Assessment of Cognition in Schizophrenia; BLERT, Bell Lysaker Emotion Recognition Task; CAI, Cognitive Assessment Interview; CANTAB, Cambridge Neuropsychological Test Automated Battery; CogState, CogState Schizophrenia Battery; DRS, Dementia Rating Scale; ER-40, Penn Emotion Recognition Task; GC, global cognition; HVLT-R, Hopkins Verbal Learning Test-Revised; LNS, Letter-Number Span; MCCB, MATRICS Consensus Cognitive Battery; NC, neurocognition; ns, non-significant; QLS, Quality of Life Scale; RCT, randomized controlled trial; RBANS, Repeatable Battery for the Assessment of Neuropsychological Status; RWF, real-world functioning; SCOPE, Social Cognition Psychometric Evaluation; SFS, Social Functioning Scale; TASIT, The Awareness of Social Inferences Test; TMT-A, Trail Making Test: Part A; US, United States; WHODAS, World Health Organization Disability Assessment Schedule.

### *Supplementary Table 8. Strength of the relationship between cognitive performance and real-world functioning across instrument pairs (n=43)*

| **Instrument pair** | | **No. studies** | **Relationship between overall instrument scores** | | | | | **Relationship between subtest/domain instrument scores*** | | | | |
| --- | --- | --- | --- | --- | --- | --- | --- | --- | --- | --- | --- | --- |
|  |  |  | **No. studies with relevant effect size data** | **Strength** | | | | **No. studies with relevant effect size data** | **Strength** | | | |
| **Cognitive performance** | **Real-world functioning** |  |  | **Minimal**  r<0.10 d<0.2 | **Small**  0.1≤r<0.3 0.20≤d<0.5 | **Medium**  0.3≤r<0.5 0.5≤d<0.8 | **Large**  r≥0.5 d≥0.8 |  | **Minimal**  r<0.10 d<0.2 | **Small**  0.1≤r<0.3 0.20≤d<0.5 | **Medium**  0.3≤r<0.5  0.5≤d<0.8 | **Large**  r≥0.5  d≥0.8 |
| MCCB | SLOF | 13^36,41,42,44,56,57,61,63,67,69,76,99,107^ | 3^57,63,76^ | 0 | 2^57,63^ | 0 | 1^76^ | 5^36,42,56,67,69^ | 2^36,67^ | 5^36,42,56,67,69^ | 3^42,67,69^ | 0 |
| BACS | QLS | 5^37,108,109,111,112^ | 4^37,108,109,111^ | 1^108^ | 1^37^ | 2^109,111^ | 0 | 0 | 0 | 0 | 0 | 0 |
| MCCB | Real-world milestones | 5^33,39,81,^  ^103,115^ | 0 | 0 | 0 | 0 | 0 | 3^33,39,103^ | 0 | 2^33,39^ | 1^103^ | 0 |
| MCCB | QLS | 4^102,104,^  ^110,113^ | 1^110^ | 0 | 1^110^ | 0 | 0 | 0 | 0 | 0 | 0 | 0 |
| MCCB | SFS | 3^40,70,97^ | 3^40,70,97^ | 1^70,97^ | 0 | 1^40^ | 0 | 0 | 0 | 0 | 0 | 0 |
| SCOPE | SLOF | 3^36,56,67^ | 0 | 0 | 0 | 0 | 0 | 3^36,56,67^ | 2^36,67^ | 3^36,56,67^ | 2^36,67^ | 0 |
| MCCB | WHODAS | 2^100,101^ | 2^100,101^ | 0 | 2^100,101^ | 0 | 0 | 0 | 0 | 0 | 0 | 0 |
| DRS | Real-world milestones | 2^58,75^ | 0 | 0 | 0 | 0 | 0 | 1^75^ | 0 | 0 | 1^75^ | 0 |
| BACS | SFS | 2^73,116^ | 1^73^ | 0 | 1^73^ | 0 | 0 | 0 | 0 | 0 | 0 | 0 |
| BACS | SLOF | 1^73^ | 1^73^ | 0 | 0 | 0 | 1^73^ | 0 | 0 | 0 | 0 | 0 |

*Individual studies can contribute to more than 1 category if multiple domains are assessed in a single instrument.

Instrument pairs with no effect size data for comparisons: BACS and real-world milestones, BACS and WHODAS, MCCB and SAS, RBANS and real-world milestones, RBANS and SLOF.

BACS, Brief Assessment of Cognition in Schizophrenia; CAI, Cognitive Assessment Interview; DRS, Dementia Rating Scale; MCCB, MATRICS Consensus Cognitive Battery; QLS, Quality of Life Scale; RBANS, Repeatable Battery for the Assessment of Neuropsychological Status; SAS, Simpson-Angus Scale; SCOPE, Social Cognition Psychometric Evaluation; SFS, Social Functioning Scale; SLOF, Specific Levels of Functioning Scale; WHODAS, World Health Organization Disability Assessment Schedule.

### *Supplementary Table 9. Characteristics and key results of final included studies examining the relationship between functional capacity and real-world functioning (n=41)*

| **Author** | **Publication Year** | **Country  (stated or clear from authorship)** | **Study design** | **Sample size^a^** | **Instrument pair(s)** | **Subtest/domain score (overall/total score not reported)** | **Key results** | **Interpretation of association** | | |
| --- | --- | --- | --- | --- | --- | --- | --- | --- | --- | --- |
|  |  |  |  |  |  |  |  | **Significance of association** | **Effect size** | |
|  |  |  |  |  |  |  |  |  | **N, analysis** | **Strength of association** |
| **Observational studies** | | | | | | | | | | |
| Bechi et al.^37^ | 2017 | Italy | Cross-sectional | 79 | UPSA and QLS |  | UPSA-B total and QLS total (r=0.15, p=0.20) | Greater FC performance showed a non-significant positive association with greater RWF performance | 79 | Small positive relationship between UPSA-B and QLS |
| Best et al.^118^ | 2014 | Not reported | Longitudinal | 136 | UPSA and SLOF SSPA and SLOF | SLOF subscales | Baseline UPSA significantly predicted SLOF Activities at 18 months for individuals with primarily positive symptoms (B=1.19, p=0.001, 95% CI=0.52 to 1.86), or undifferentiated symptoms (B=0.78, p=0.002, 95% CI CI=0.28 to1.28). However, the relationship was not significant for primarily negative symptoms (B=-0.13, p=0.864, 95% CI=−1.61 to 1.4) or depressive symptoms (B=0.04, p=0.759, 95% CI=−0.22 to 0.31)  Baseline SSPA significantly predicted SLOF interpersonal functioning at 18 months for individuals with primarily positive symptoms (B=4.31, p<0.001, 95% CI=2.03 to -6.59), or undifferentiated symptoms (B=1.81, p=0.031, 95% CI=0.173.44). However, the relationship was not significant for primarily negative symptoms (B=0.19, p=0.940, 95% CI=−4.77 to 5.14) or depressive symptoms (B=-0.28, p=0.836, 95% CI=-2.96 to 2.40) | Greater RWF performance in activities showed a significant association with greater FC performance for patients with primarily positive or undifferentiated symptoms but not for those with primarily negative or depressive symptoms | Effect size not interpreted - only regression coefficient reported | |
| Bosgelmez et al.^119^ | 2015 | Turkey | Cross-sectional | 95 | CAI and SFS |  | CAI-TR overall score and SFS (r=-0.520, p<0.01) | Greater FC performance showed a significant association with greater RWF performance | 90 | Large negative relationship between CAI-TR overall score and SFS |
| Bowie et al.^120^ | 2010 | US | Cross-sectional | 161 | UPSA-B and SLOF SSPA and SLOF | SLOF subscales | Adaptive competence (UPSA) directly predicted SLOF activities (β=0.35) Social competence (SSPA) directly predicted SLOF work skills (β=0.21) Social competence (SSPA) directly predicted SLOF interpersonal behaviour (β=0.26) | Greater RWF activities performance directly was predicted by greater adaptive competence performance but not social competence  Greater RWF work skills performance and interpersonal behavior directly predicted by greater social competence performance but not adaptive competence | Effect size not interpreted - only regression coefficient value reported | |
| Bowie et al.^121^ | 2008 | US | Cross-sectional | 222 | UPSA and SLOF SSPA and SLOF | SLOF subscales | Interpersonal behaviour directly predicted by social competence (SSPA) (β=0.16) but not functional competence (UPSA) Community activity participation directly predicted by social competence (SSPA) (β=0.24) and functional competence (UPSA) Work skills directly predicted by social competence (SSPA) (β=0.22) and functional competence (UPSA) (β=0.12) | Greater RWF interpersonal behavior performance was directly predicted by greater social competence performance but not adaptive/functional competence  Greater RWF community activity participation directly predicted by greater social competence and adaptive/functional competence performance  Greater RWF work skills performance directly predicted by greater social competence and adaptive/functional competence | Effect size not interpreted - only regression coefficient value reported | |
| Bowie et al.^122^ | 2007 | US | Cross-sectional | 67 | UPSA and SLOF SSPA and SLOF | SLOF subscales | SLOF domains (case manager-/self-rated) and UPSA: interpersonal domain (r=0.17, ns/r=-0.08, ns), activities domain (r=0.42, p<0.001/r=0.15, ns), work domain (r=0.29, ns/r=0.16, ns) SLOF domains (case manager-/self-rated) and SSPA: interpersonal domain (r=0.51, p<0.001/r=0.36, ns), activities domain (r=0.26, ns/r=0.15, ns), work domain (r=0.38, ns/r=0.29, ns) | Adaptive/functional competence: Greater case manager and self-rated RWF (interpersonal relationships) showed a non-significant association with greater FC; greater case manager-rated RWF (activities) showed a significant association with greater FC but a non-significant association for self-rated RWF; greater case manager-rated and self-rated RWF (work skills) showed a non-significant association with greater FC  Social competence: Greater case manager-rated RWF (interpersonal relationships) showed a significant association with greater FC. Self-rated showed a non-significant association with greater FC; greater case manager-rated RWF (activities) showed a non-significant association with greater FC; greater case manager-rated RWF (work skills) showed a non-significant association with greater FC | 67 | Small positive relationship between SLOF interpersonal domain and UPSA (manager-rated),  minimal negative relationship between SLOF interpersonal domain and UPSA (self-rated), SLOF work domain (both case manager- and self-rated) and UPSA; small positive relationship between SLOF activities domain (self-rated) and UPSA; medium positive relationship between SLOF activities domain (case manager-rated) and UPSA  Small positive relationship between SLOF activities domain (both case manager-/self-rated) and SSPA; small positive relationship between SLOF work domain (self-rated) and SSPA; medium positive relationship between SLOF interpersonal domain (self-rated) and SSPA and between SLOF work domain (case manager-rated) and SSPA; large positive relationship between SLOF interpersonal domain (case manager-rated) and SSPA |
| Cardenas et al.^123^ | 2013 | Not reported | Cross-sectional | 97 | UPSA-B and QLS |  | UPSA was significantly related to QLS when self-efficacy was high (B=0.242 ±0.102, p=0.020) but not low (B=-0.039 ±0.110, p=0.727) | Greater FC performance was significantly related to greater RWF when self-efficacy was high but not when low | Effect size not interpreted - only regression coefficient value reported | |
| Cruz et al.^124^ | 2016 | Brazil | Cross-sectional | 79 | SCoRS and QLS |  | SCoRS was a significant predictor of QLS (r_s_=0.471, p<0.001) | Greater FC performance showed a significant association with greater RWF performance | 79 | Medium negative relationship between SCoRS and QLS |
| Fujino et al.^40^ | 2016 | Japan | Cross-sectional | 93 | UPSA-B and SFS |  | UPSA-B significantly predicted SFS total scores (r=0.389, p<0.01) UPSA-B significantly predicted SFS employment domain (r=0.486, p<0.01) | Greater FC performance showed a significant association with greater RWF performance | 93 | Medium positive relationship between UPSA-B and SFS total score; medium positive relationship between UPSA-B and SFS employment domain |
| Galderisi et al.^41^ | 2016 | Italy | Cross-sectional | 921 | UPSA-B and SLOF | SLOF subscales | UPSA-B showed significant direct effects on SLOF work skills (β=0.136, p<0.001) | Greater FC showed a significant direct association with greater RWF performance in work skills but not RWF interpersonal relationships | Effect size not interpreted - only regression coefficient value reported | |
| Giordano et al.^42^ | 2022 | Italy | Cross-sectional | 580 | CAI and SLOF | SLOF subscales | CAI composite global score and SLOF scales: Interpersonal relationship (r=-0.303, p<0.0004), Everyday life skills (r=-0.507, p<0.0004), Working skills (r=-0.487, p<0.0004) | Greater FC performance showed a significant association with greater RWF interpersonal relationship and a significant association with greater RWF everyday life skills and RWF working skills | 580 | Medium negative relationship between CAI composite global score and SLOF interpersonal relationship scale; large negative relationship between CAI composite global score and SLOF everyday life skills scale; medium negative relationship between CAI composite global score and SLOF working skills scale |
| Gould et al.^99^ | 2015 | US | Cross-sectional | 214 | UPSA-B and SLOF CAI and SLOF | SLOF subscales | UPSA-B total and SLOF subscales: Interpersonal (r=0.26, p<0.01), Activities (r=0.28, p<0.01), Vocational (r=0.31, p<0.01) Predictive value of UPSA-B for SLOF scales in step-wise regression: Interpersonal function, step 2 R^2^=0.17, p<0.001; Vocational, step 1 R^2^=0.11, p<0.001 CAI composite global score and SLOF subscales: Interpersonal (r=-0.06, ns), Activities (r=-0.28, p<0.01), Vocational (r=-0.42, p<0.01) | Greater FC showed a significant association with greater RWF  [CAI ns for interpersonal relationships] | 214 | Small positive relationship between UPSA-B total and SLOF interpersonal subscale; small positive relationship between UPSA-B total and SLOF activities subscale; medium positive relationship between UPSA-B total and SLOF vocational subscale. Small negative relationship between CAI composite global and SLOF interpersonal subscale; small negative relationship between CAI composite global and SLOF activities subscale; medium negative relationship between CAI composite global and SLOF vocational subscale Effect size not interpreted for UPSA-B/SLOF step-wise regression (r squared values) |
| Green et al.^30^ | 2011 | US | Cross-sectional | 166 | UPSA and QLS CAI and QLS |  | QLS and UPSA (r=0.25), and UPSA short form (r=0.15) QLS and CAI (r=0.27) [p-values not reported] | Greater FC performance showed an association with greater RWF performance | 163 | Small positive relationship between QLS and UPSA; small positive relationship between QLS and CAI |
| Green et al.^43^ | 2008 | US | Cross-sectional | 176 | UPSA and global functional outcome (SFS and SAS) MASC and global functional outcome |  | MASC and global functional outcome (data combined across sites)/(median 5 sites): conversational content (r=0.28/0.28), nonverbal content (r=0.23/0.20), effectiveness (r=0.27/0.28) UPSA Total score and global functional outcome (data combined across sites)/(median r of 5 sites): r=0.23/0.29 SCoRS (interviewer) and global functional outcome (data combined across sites)/(median r of 5 sites): r=-0.34/-0.37 [p-values not reported] | Greater FC performance showed an association with greater RWF performance | 156 | Small positive relationship between MASC conversational content/MASC non-verbal content/MASC effectiveness and global functional outcome Small positive relationship between UPSA total score and global functional outcome Medium negative relationship between SCoRS/interviewer and global functional outcome |
| Hajdúk et al.^44^ | 2021 | US | Cross-sectional | 408 | UPSA-B and SLOF SSPA and SLOF |  | FC showed a strong direct relationship with RWF | Greater FC showed a direct association with greater RWF performance | Effect size not interpreted - network analysis | |
| Harvey et al.^133^ | 2011 | US | Cross-sectional | 198 | UPSA-B and SLOF |  | SLOF established as superior predictor of performance-based measures. SLOF total score was significantly related to the ability trait, t(192)=3.09, p=0.002 | Greater FC was significantly associated with greater RWF | Effect size not interpreted - regression analysis | |
| Ho et al.^46^ | 2013 | US | Cross-sectional | 138 | UPSA and SLOF | SLOF subscales | UPSA significantly predicted SLOF activities (B=0.128 ±0.055, p<0.05) UPSA significantly predicted SLOF work skills (B=0.069 ±0.034, p<0.05) | FC performance significantly predicted RWF performance in everyday activities and work skills but not other SLOF domains | Effect size not interpreted - only regression coefficient value reported | |
| Iasevoli et al.^125^ | 2018 | Italy | Cross-sectional | 60 | UPSA extended version and SLOF | SLOF subscales | UPSA and SLOF scales, in TRS patients: physical functioning (r=0.66, p<0.0005), personal care skills (r=0.43, p=0.21), interpersonal relationships (r=0.53, p=0.004), social acceptability (r=0.43, p=0.21), community living (r=0.61, p=0.001), work skills (r=0.61, p=0.005) UPSA and SLOF scales, in non-TRS patients, p>0.05 for all scales [no r values reported] | In patients with treatment-resistant schizophrenia, greater FC showed a significant association with greater RWF interpersonal relationship; a significant association with RWF physical functioning, greater community living and work skills; a non-significant association with RWF personal care skills and social acceptability. No significant associations were shown for patients without treatment-resistant schizophrenia | 28 | Large positive relationship between UPSA and SLOF physical functioning, interpersonal relationships, community living, and work skills scales Medium positive relationship between UPSA and SLOF personal care skills, and acceptability scales All in patients with treatment resistant schizophrenia (TRS) |
| Kang et al.^126^ | 2017 | Korea | Cross-sectional | 84 | SCoRS and SFS |  | K-SCoRS (global rating) and SFS Total (r=-0.110, p=0.313) | Greater FC performance showed a non-significant association with RWF performance | 84 | Small negative relationship between K-SCoRS (global rating) and SFS Total |
| Keefe et al.^52^ | 2016 | US | Cross-sectional | 167 | UPSA and SLOF VRFCAT and SLOF  SCoRS and SLOF |  | UPSA-VIM and SLOF (r=0.25, p<0.01) SLOF and VRFCAT, Total time (r=0.22, p<0.01), Total Errors (r=0.29, p<0.001), Progression (r=0.17, p<0.05) SCoRS and SLOF (r=0.57, p<0.001) | Greater FC showed a significant association with greater RWF | 158 | Large positive relationship between SCoRS and SLOF  Small positive relationship between UPSA-VIM and SLOF Small positive relationship between SLOF and VRFCAT, Total time, Total Errors, Progression |
| Kim et al.^100^ | 2021 | Korea | Cross-sectional | 49 | UPSA and WHODAS |  | UPSA total score and WHODAS overall score (r=-0.254, ns) | Greater FC showed a non-significant association with greater RWF performance | 49 | Small negative relationship between UPSA total score and WHODAS overall score |
| Lindenmayer et al.^127^ | 2020 | US | Longitudinal | 62 | VRFCAT and SLOF |  | VRFCAT composite score (baseline) and SLOF total score (baseline) (r_s_ =0.10, p=0.45) In linear regression, VRFCAT composite score at baseline was not significantly associated with the overall SLOF at 4 weeks | Greater FC performance showed a non-significant association with greater RWF performance | 62 | Small positive relationship between VRFCAT composite score (baseline) and SLOF total score (baseline) |
| Mausbach et al.^58^ | 2008 | US | Cross-sectional | 434 | UPSA and real-world milestones | Milestone (living status) | UPSA was an adequate predictor of independent living status (ROC AUC= 0.74; 95% CI: 0.68–0.79) | FC performance was an adequate predictor of independent living status | Effect size not interpreted - only ROC AUC value reported | |
| Miller et al.^33^ | 2021 | US | Cross-sectional | 557 | UPSA-B and real-world milestones SSPA and real-world milestones | Milestone (living status and financial independence) | UPSA-B and residential independence (Y/N) : t=1.82, p=0.066, Cohen’s d=0.23^b^  SSPA and residential independence (Y/N): t=2.30, p=0.026, Cohen’s d=0.28^b^   UPSA-B and financial independence (Y/N): t=4.29, p<0.001, Cohen’s d=0.40^b^  SSPA and financial independence (Y/N): t=2.56, p=0.01, Cohen’s d=0.24^b^ | Greater FC showed a significant association with greater RWF performance | 480 | Small difference in between UPSA-B by residential independence status (Y/N) Small difference in SSPA by residential independence status (Y/N) Small difference in UPSA-B by financial independence status (Y/N) Small difference in SSPA by financial independence status (Y/N) |
| Mucci et al.^61^ | 2021 | Italy | Longitudinal | 618 | UPSA-B and SLOF | SLOF subscales | Higher baseline SLOF everyday life skills were associated, as measured in the model by standardized regression coefficients, with positive change at 4-year follow-up in UPSA-B total score (β=0.508; 95% CI, 0.331 to 0.685; p<0.001) | FC performance showed a significant positive association with RWF performance in everyday life skills but not interpersonal relations or work skills | Effect size not interpreted - only regression coefficient value reported | |
| Nagendra et al.^63^ | 2020 | US | Cross-sectional | 169 | UPSA and SLOF  SSPA and SLOF |  | UPSA-B and SLOF informant rating (r=0.19, p<0.05) SSPA and SLOF informant rating: r=0.23, p<0.01) | FC performance showed a significant positive association with RWF informant-rated performance | 169 | Small positive relationship between UPSA-B and SLOF informant rating Small positive relationship between SSPA and SLOF informant rating |
| Okada et al.^134^ | 2021 | Japan | Cross-sectional | 107 | SCoRS and SFS | SFS domains | SCoRS total score was significantly correlated with all 7 SFS domains: withdrawal (r=-0.305, p<0.01), interpersonal relationships (r=-0.357, p<0.01), social participation (r=-0.570, p<0.01), recreation (r=-0.443, p<0.01), self-reliance and ability (r=-0.658, p<0.01), self-reliance and execution (r=-0.363, p<0.01) and employment (r=-0.612, p<0.01) | Greater FC performance showed a significant association with greater RWF performance | 107 | Large negative relationship between SCoRS total score and self-reliance and ability domain Large negative relationship between SCoRS total score and social participation domain Medium negative relationship between SCoRS total score and recreation domain Large negative relationship between SCoRS total score and employment domain Medium negative relationship between SCoRS total score and withdrawal domain Medium negative relationship between SCoRS total score and self-reliance and execution domain Medium negative relationship between SCoRS total score and interpersonal relationships domain |
| Okada et al.^128^ | 2020 | Japan | Cross-sectional | 107 | SCoRS and SFS |  | SCoRS and SFS (r=0.565, p <0.01) Modelling concluded that neurocognitive function (SCoRS) significantly affects real world functioning (SFS) independent of expression and experience | Greater FC performance showed a significant association with greater RWF performance | 107 | Large positive relationship between SCoRS and SFS |
| Olsson et al.^129^ | 2016 | Sweden | Cross-sectional | 235 | UPSA-B and real-world milestones | Milestones (education, living status, marital status, social contacts) | Education level was significantly associated with UPSA-B. An increase in FC (higher UPSA-B scores) was significantly associated with increased odds of higher education level (OR: 1.036, 95% CI: 1.019–1.054, p<0.001) Housing situation was significantly associated with UPSA-B. Patients with lower FC (lower UPSA-B scores) was significantly associated with increased odds of living in supported housing (OR: 1.033, 95% CI: 1.011–1.056, p=0.003) No significant association was shown for social contacts and marital status | Greater FC performance showed a significant association with increased odds of higher education  Lower FC performance showed a significant association with increased odds of living in supported housing. No significant association was shown with social contacts or marital status | Effect size not interpreted - only OR reported | |
| Patterson et al.^65^ | 2001 | US | Cross-sectional | 83 | SSPA and SAS |  | SSPA total score and SAS-Global functioning (r=-0.17, ns) | Greater FC performance showed a non-significant association with RWF performance | 83 | Small negative relationship between SSPA total score and SAS-Global functioning |
| Reichenberg et al.^130^ | 2014 | Not reported | Longitudinal | 111 | UPSA and SSPA |  | UPSA and SSPA scores had significant effects on SLOF total score (UPSA: χ2=12.85, p<0.001; SSPA: χ2=5.26, p<0.005) Lower UPSA and SSPA scores were significant predictors of worsening SLOF everyday functioning (UPSA: χ2=24.01, p<0.001; SSPA: χ2=4.83, p=0.03) and vocational functioning (UPSA: χ2=6.51, p<0.025; SSPA: χ2=4.60, p=0.04) but not social functioning or everyday activities | Greater FC showed a significant association with greater RWF | Effect size not interpreted - only χ2 value reported | |
| Stergiopoulos et al.^131^ | 2011 | Canada | Case control | 51 | UPSA and real-world milestones | Milestone (living status) | There was not a significant difference between homeless and housed participants in the total UPSA score (χ2=3.12, p=0.07) There was not a significant difference between homeless and housed participants in all UPSA subscales except the financial subscale (p<0.05) | FC did not show a significant association with homelessness | Effect size not interpreted - only regression coefficient and χ2 reported | |
| Sumiyoshi et al.^73^ | 2016 | Japan | Cross-sectional | 58 | UPSA and SLOF UPSA and SFS |  | UPSA-B and SLOF (r=0.654, p<0.01) UPSA-B and SFS (r=0.342, p<0.01) | FC performance showed a significant positive association with RWF performance | 58 | Large positive relationship between UPSA-B and SLOF Medium positive relationship between UPSA-B and SFS |
| Takahashi et al.^108^ | 2017 | Japan | Cross-sectional | 45 | SCoRS and QLS |  | QLS score and SCoRS global rating score (r=-0.45, p=0.002) | Greater FC performance showed a significant association with greater RWF performance | 45 | Medium negative relationship between QLS score and SCoRS global rating score |
| Twamley et al.^75^ | 2002 | US | Cross-sectional | 111 | UPSA and real-world milestones |  | UPSA total score was positively correlated with the degree of independence in the community living situation (rs=0.48, p<0.001). | Greater FC performance showed a significant association with greater RWF performance | 111 | Medium positive relationship between UPSA total score and the degree of independence in the community living situation |
| Velligan et al.^76^ | 2014 | India | Cross-sectional | 141 | UPSA-B and SLOF |  | UPSA-B and SLOF (r=0.13, ns) | FC performance showed a non-significant positive association with RWF performance | 141 | Small positive relationship between UPSA and SLOF |
| Ventura et al.^77^ | 2010 | US | Cross-sectional | 176 | SCoRS and SFS CAI and SFS |  | SCoRS and SFS (patient/informant/rater): (r=-0.23/-0.24/-0.34, all ns) CAI and SFS (patient/informant/rater): (r=-0.31/-0.27/-0.32, all p<0.01) | FC performance showed a significant weak association with overall RWF [ns for SCoRS] | 172 | Small negative relationship between Birchwood Social Functioning Scale and SCoRS (patient/informant) Medium negative relationship between Birchwood Social Functioning Scale and SCoRS (rater composite) Medium negative relationship between Birchwood Social Functioning Scale and CAI (patient/rater composite) Small negative relationship between Birchwood Social Functioning Scale and CAI (informant) |
| Vesterager et al.^81^ | 2012 | Denmark | Longitudinal | 117 | UPSA-B and real-world milestones |  | Functional capacity was only modestly associated with real-world measures of functioning. Functional capacity significantly predicted to global functioning in the univariate analysis (B=0.04, p<0.01), but in multivariable analyses global functioning, financial independence, and independent living were only significantly predicted by negative symptoms or general symptoms | FC was only modestly associated with RWF performance | Effect size not interpreted - only regression coefficient value reported | |
| Wright et al.^132^ | 2020 | United Kingdom | Cross-sectional | 52 | UPSA and real-world milestones | Milestone (employment) | UPSA did not significantly predict employment status (p=0.07)  UPSA total did not predict number of hours worked (p>0.05) | Greater FC did not show a significant association with employment | Effect size not interpreted - no r value | |
| **Interventional studies** | | | | | | | | | | |
| Gupta et al.^135^ | 2012 | Not reported | Other interventional | 54 | UPSA and SLOF SSPA and SLOF MMAA and SLOF |  | SSPA total score positively correlated with SLOF interpersonal (r=0.36, p=0.003), SLOF activities (r=0.19, p=0.08) and SLOF work (r=0.12, p=0.19) UPSA total score positively correlated with SLOF activities (r=0.38, p=0.002), SLOF IP (r=0.15, p=0.13) and SLOF work (r=0.20, p=0.07) MMAA was positively correlated with SLOF IP (r=0.21, p=0.06), SLOF activities (r=0.17, p=0.10) and SLOF work (r=0.12, p=0.18) | Greater FC showed a significant weak association with greater RWF interpersonal relationships [SSPA only] and everyday activities [UPSA only] | 54 | Medium positive relationship between SSPA total score and SLOF interpersonal  Small positive relationship between SSPA total score and SLOF activities Small positive relationship between SSPA total score and SLOF work Medium positive relationship between UPSA total score and SLOF activities  Small positive relationship between UPSA total score and SLOF IP Small positive relationship between UPSA total score and SLOF work Small positive relationship between MMAA and SLOF work Small positive relationship between MMAA and SLOF IP Small positive relationship between MMAA and SLOF activities |
| Twamley et al.^136^ | 2012 | US | RCT | 30 | UPSA and real-world milestones | Milestone (employment) | UPSA total score and attainment/week/wage of competitive work in the group receiving conventional vocational rehabilitation: r=0.10, p=0.665/r_s_=0.042, p=0.854/r_s_=0.06, p=0.793 UPSA total score and attainment/week/wage of competitive work in the group receiving individual placement and support: r=0.41, p=0.041/r_s_=0.37, p=0.072/r_s_=0.32, p=0.115 | Greater FC showed a non-significant association with greater attainment/week/wage of competitive work Individual placement and support group work attainment showed a significant association with greater FC performance | Conventional vocational rehabilitation group, n=28; individual placement and support group, n=30 | Group receiving conventional vocational rehabilitation: Small positive relationship between UPSA total score and attainment of competitive work Minimal positive relationship between UPSA total score and week/wage of competitive work  Group receiving individual placement and support: Medium positive relationship between UPSA total score and attainment/week/wage of competitive work |

^a^Number of individuals with a diagnosis of schizophrenia, schizophrenia spectrum disorders, and first episode non-affective psychosis.

^b^Analysis of unpublished data obtained directly from the study authors.

FC, functional capacity; MASC, Maryland Assessment of Social Competence; MMAA, Multimodal Attention Assessment Battery; QLS, Quality of Life Scale; RCT, randomized controlled trial; RWF, real-world functioning; SAS, Simpson-Angus Scale; SCoRS, Schizophrenia Cognition Rating Scale; SCZ, schizophrenia; SFS, Social Functioning Scale; SLOF, Specific Level of Functioning scale; SSPA, Social Skills Performance Assessment; UPSA, UCSD Performance-Based Skills Assessment; UPSA-B, UCSD Performance-Based Skills Assessment - Brief version; US, United States; WHODAS, World Health Organization Disability Assessment Schedule.

### *Supplementary Table 10. Strength of the relationship between functional capacity and real-world functioning across instrument pairs (n=41)*

| **Instrument pair** | | **No. studies** | **Relationship between overall instrument scores** | | | | | **Relationship between subtest/domain instrument scores*** | | | | |
| --- | --- | --- | --- | --- | --- | --- | --- | --- | --- | --- | --- | --- |
|  |  |  | **No. studies with relevant effect size data** | **Strength** | | | | **No. studies with relevant effect size data** | **Strength** | | | |
| **Functional capacity** | **Real-world functioning** |  |  | **Minimal**  r<0.10 d<0.2 | **Small**  0.1≤r<0.3 0.20≤d<0.5 | **Medium**  0.3≤r<0.5 0.5≤d<0.8 | **Large**  r≥0.5 d≥0.8 |  | **Minimal**  r<0.10  d<0.2 | **Small**  0.1≤r<0.3 0.20≤d<0.5 | **Medium**  0.3≤r<0.5  0.5≤d<0.8 | **Large**  r≥0.5  d≥0.8 |
| UPSA/UPSA-B | SLOF | 16^41,44,46,52,^  ^61,63,73,76,99,^  ^118,120–122,125,^  ^133,135^ | 5^52,63,73,76,135^ | 0 | 4^52,63,76,135^ | 1^135^ | 1^73^ | 3^99,122,125^ | 1^122^ | 2^99,122^ | 3^99,122,125^ | 1^125^ |
| UPSA/UPSA-B | Real-world milestones | 8^33,58,75,81,129,131,132,136^ | 0 | 0 | 0 | 0 | 0 | 3^33,75,136^ | 1^136^ | 1^33,136^ | 1^75,136^ | 0 |
| SSPA | SLOF | 7^44,63,118,120–122,135^ | 2^63,135^ | 0 | 2^63,135^ | 0 | 0 | 1^122^ | 0 | 1^122^ | 1^122^ | 1^122^ |
| SCoRS | SFS | 4^77,126,128,134^ | 3^77,126,128^ | 0 | 2^77,126^ | 0 | 1^128^ | 1^134^ | 0 | 0 | 1^134^ | 1^134^ |
| UPSA/UPSA-B | QLS | 3^30,37,123^ | 2^30,37^ | 0 | 2^30,37^ | 0 | 0 | 0 | 0 | 0 | 0 | 0 |
| CAI | SFS | 2^77,119^ | 2^77,119^ | 0 | 1^77^ | 1^77^ | 1^119^ | 0 | 0 | 0 | 0 | 0 |
| CAI | SLOF | 2^42,99^ | 0 | 0 | 0 | 0 | 0 | 2^42,99^ | 0 | 1^99^ | 1^42,99^ | 1^42^ |
| SCoRS | QLS | 2^108,124^ | 2^108,124^ | 0 | 0 | 2^108,124^ | 0 | 0 | 0 | 0 | 0 | 0 |
| UPSA/UPSA-B | SFS | 2^40,73^ | 2^40,73^ | 0 | 0 | 2^40,73^ | 0 | 0 | 0 | 0 | 0 | 0 |
| VRFCAT | SLOF | 2^52,127^ | 2^52,127^ | 0 | 2^52,127^ | 0 | 0 | 0 | 0 | 0 | 0 | 0 |
| MASC | Global functional outcome | 1^43^ | 1^43^ | 0 | 1^43^ | 0 | 0 | 0 | 0 | 0 | 0 | 0 |
| CAI | QLS | 1^30^ | 1^30^ | 0 | 1^30^ | 0 | 0 | 0 | 0 | 0 | 0 | 0 |
| SCoRS | SLOF | 1^52^ | 1^52^ | 0 | 0 | 0 | 1^52^ | 0 | 0 | 0 | 0 | 0 |
| SSPA | Real-world milestones | 1^33^ | 0 | 0 | 0 | 0 | 0 | 1^33^ | 0 | 1^33^ | 0 | 0 |
| SSPA | SAS | 1^65^ | 1^65^ | 0 | 1^65^ | 0 | 0 | 0 | 0 | 0 | 0 | 0 |
| UPSA | Global functional outcome | 1^43^ | 1^43^ | 0 | 1^43^ | 0 | 0 | 0 | 0 | 0 | 0 | 0 |
| UPSA | WHODAS | 1^100^ | 1^100^ | 0 | 1^100^ | 0 | 0 | 0 | 0 | 0 | 0 | 0 |
| MMAA | SLOF | 1^135^ | 1^135^ | 0 | 1^135^ | 0 | 0 | 0 | 0 | 0 | 0 | 0 |

*Individual studies can contribute to more than 1 category if multiple domains are assessed in a single instrument.

Instrument pairs with no effect size data for comparisons: UPSA and SSPA.

CAI, Cognitive Assessment Interview; MASC, Maryland Assessment of Social Competence; MMAA, Multimodal Attention Assessment Battery; QLS, Quality of Life Scale; SAS, Simpson-Angus Scale; SCoRS, Schizophrenia Cognition Rating Scale; SFS, Social Functioning Scale; SLOF, Specific Level of Functioning scale; SSPA, Social Skills Performance Assessment; UPSA, UCSD Performance-Based Skills Assessment; UPSA-B, UCSD Performance-Based Skills Assessment - Brief version; VRFCAT, Virtual Reality Functional Capacity Assessment Tool; WHODAS, World Health Organization Disability Assessment Schedule.
